# Evolutionary prediction of medicinal properties in the genus *Euphorbia* L.

**DOI:** 10.1038/srep30531

**Published:** 2016-07-28

**Authors:** Madeleine Ernst, C. Haris Saslis-Lagoudakis, Olwen M. Grace, Niclas Nilsson, Henrik Toft Simonsen, James W. Horn, Nina Rønsted

**Affiliations:** 1 Natural History Museum of Denmark, Faculty of Science, University of Copenhagen, Sølvgade 83S, DK-1307 Copenhagen K, Denmark; 2 Comparative Plant & Fungal Biology, Royal Botanic Gardens, Kew, Surrey TW9 3AB, United Kingdom; 3 Skin Research, LEO Pharma A/S, Industriparken 55, DK-2750 Ballerup, Denmark; 4 Department of Systems Biology, Technical University of Denmark, DK-2800 Kgs. Lyngby, Denmark; 5 Natural and Applied Sciences, University of Wisconsin, Green Bay, LS 458, UW-Green Bay, 2420 Nicolet Dr, Green Bay, WI 54311-7001, USA

## Abstract

The current decrease of new drugs brought to the market has fostered renewed interest in plant-based drug discovery. Given the alarming rate of biodiversity loss, systematic methodologies in finding new plant-derived drugs are urgently needed. Medicinal uses of plants were proposed as proxy for bioactivity, and phylogenetic patterns in medicinal plant uses have suggested that phylogeny can be used as predictive tool. However, the common practice of grouping medicinal plant uses into standardised categories may restrict the relevance of phylogenetic predictions. Standardised categories are mostly associated to systems of the human body and only poorly reflect biological responses to the treatment. Here we show that medicinal plant uses interpreted from a perspective of a biological response can reveal different phylogenetic patterns of presumed underlying bioactivity compared to standardised methods of medicinal plant use classification. In the cosmopolitan and pharmaceutically highly relevant genus *Euphorbia* L., identifying plant uses modulating the inflammatory response highlighted a greater phylogenetic diversity and number of potentially promising species than standardised categories. Our interpretation of medicinal plant uses may therefore allow for a more targeted approach for future phylogeny-guided drug discovery at an early screening stage, which will likely result in higher discovery rates of novel chemistry with functional biological activity.

Plants have played a central role in human health-care since ancient times^1^,^2^. Although the plant domain is widely regarded as natural capital with potential to yield new drugs, this potential is under threat due to the alarming biodiversity loss, with recent estimates indicating that every fifth plant species on earth is threatened with extinction^3^. Unlocking the potential of plants in health-care therefore urges for a time-efficient and systematic approach.

Following the assumption that plant-derived chemicals are constrained to evolutionary plant lineages^4^,^5^,^6^, phylogeny-guided approaches have been seen as one of the time-efficient and informed approaches to plant-based drug discovery^7^,^8^,^9^,^10^,^11^,^12^,^13^,^14^. Some of these approaches utilise information from ethnomedicine: the use of plants by humans as medicines^15^. Reports of medicinal plant uses are employed as a proxy for bioactivity and are superimposed on phylogenetic trees e.g.^8^,^11^,^14^. Evolutionary methodologies then predict potential bioactivity of different plant lineages, based on the distribution of medicinal plant use across the phylogeny.

One potential limitation of the phylogenetic approach lies in the classification of medicinal plant use. Prior to being analysed in a phylogenetic context, documented plant medicinal uses are collected and classified according to the diseases they are used to treat as typically done in ethnomedicinal research^16^. For this purpose, both internationally recognised medical standards such as the International Classification of Diseases (ICD; http://www.who.int/classifications/icd/en/) of the WHO, as well as a classification system developed in the field of Economic Botany (Economic Botany Data Collection Standard, EBDCS^17^) are widely used^16^. Although these classification systems are useful to guarantee consistency, data exchange and comparability among different studies^16^,^18^, they have two major drawbacks. First, they do not fully capture the complexity and idiosyncrasy of local plant-based healthcare^6^,^19^,^20^. Second, and most important, they are based on categories reflecting systems of the human body (e.g. digestive system) or symptoms. Not only are classifications based on systems of the body affected or common symptoms little informative for disease etiology^21^, but they also allow very little insight into the potential underlying biological activity of the medicinal plants. In recent years cellular and molecular mechanisms underlying diseases have been extensively studied^22^ and aided the discovery of disease etiology. We postulate that in a phylogenetic context a classification based on a biological response provoked by the treatment may reflect more accurately the cellular or molecular mechanisms underlying the disease’s treatment and thus provides a more accurate proxy of the biological activities of the plant-derived compounds and therefore enables a more accurate phylogenetic prediction of distinct biological activities triggered by the plants (Fig. 1).

Here, we investigate the influence of medicinal plant use classification in a phylogenetic context, using the genus *Euphorbia* L. (Euphorbiaceae) as an example. With about 2,000 recognized species^23^,^24^, *Euphorbia* is among the three largest genera of angiosperms, with a near-cosmopolitan distribution and remarkable morphological diversity, including annual herbs, succulents and large trees, united by a unique, flower-like inflorescence and often poisonous, milky latex^24^,^25^. The molecular phylogenetics of the genus has been extensively studied^24^,^26^,^27^,^28^,^29^,^30^ and a large compendium of data on the medicinal uses is available^31^. The diterpenoid ingenol mebutate, a chemical compound isolated from *Euphorbia peplus* L. is marketed as a drug for the treatment of actinic keratosis, a precancerous skin condition^32^. Nevertheless ingenol mebutate is only obtained in extremely low quantities from the plant, making its production inefficient^33^. Alternative sources are therefore desired and investigated^33^. *Euphorbia* exemplifies the need for a systematic approach to plant-based drug discovery: with approximately 5% of species in the genus chemically investigated^34^, how do we go about prioritizing which of the remaining 1,900 species to investigate?

We follow the core hypothesis that phylogenetic patterns in medicinal properties are underlied by similarities in phytochemical properties. We apply two interpretative approaches to the medicinal uses of *Euphorbia* – one following a standard classification system used in ethnomedicinal studies, the other aiming to identify plant uses modulating the inflammatory response. Inflammatory processes are highly relevant in the treatment of actinic keratosis by ingenol mebutate^32^. We scrutinize phylogenetic patterns in species recovered by both approaches and describe the influence of classification of medicinal plant use on the *Euphorbia* species identified for potential early drug discovery by selected phylogenetic measures. We refer to medicinal properties as properties described from ethnomedicinal research^31^. Data presented in this study are exclusively based on ethnomedicinal use reports of species of *Euphorbia*^31^ and do not include new chemical data on the genus *Euphorbia*. Instead, we refer to two extensive reviews on the chemical and pharmacological properties of species of the genus *Euphorbia*^34^,^35^. The context for the data presented in this study is therefore an assessment of an *in vitro* early stage screening in drug discovery, before parameters such as drugability, safety, formulation or comparative effectiveness of isolated compounds are considered.

## Results

We classified ethnomedicinal data using two different approaches: one that is used commonly in ethnomedicinal studies and one based on the biological response to the treatment. In specific, we identified plant uses modulating the inflammatory response. Inflammation is a prominent biological response to ingenol mebutate, which plays an important role in the treatment of actinic keratosis^32^,^36^. The first approach (EBDCS) is widely used in ethnomedicinal studies^18^ and classifies uses of plants into standardized descriptors and terms in hierarchical level states^17^. Level 1 states are subdivided into 13 categories (e.g. *food*, *materials*, *fuels*, *medicines*, *vertebrate poisons* etc.), whereas level 2 states break down level 1 states in more detail^17^. The level 2 states of the use category *medicines* described by the EBDCS are mostly linked to systems of the body or symptoms of a disease, allowing only little insight into the disease’s cause or assumptions on a distinct biological activity or chemical nature of the medicinal plants. On contrary, our interpretation of plant medicinal uses gives a better insight into how the plant’s chemicals might interfere in the disease process (pharmacological effect^37^) and thus is a better proxy for the plant’s biological activity. Plant uses identified as modulating the inflammatory response were collected within a category we refer to as *inflammatory response.* We compared the category *inflammatory response* with the EBDCS categories investigating the effect of data organisation on evolutionary patterns relevant to early drug discovery.

First, using publicly available data, we produced the most comprehensive phylogenetic hypothesis of *Euphorbia* to date, including 560 *Euphorbia* species (>25% of the genus) representing all known subgeneric clades. In agreement with previous studies^24^,^30^ our topology (Supplementary Figure 1) confirms the presence of four subgeneric clades with posterior probability (PP) branch support values > 0.99 (except for subgenus *Esula sensu lato* PP = 0.90; subgenus *Esula* excluding *E. lathyris, E. lagascae* and *E. phymatosperma* PP = 0.99). *Euphorbia* subg. *Esula* (mainly herbaceous species with centre of diversity in temperate Europe^29^) is sister to the three remaining subgenera. *Euphorbia* subg. *Athymalus* (highly diverse succulent species with predominantly African distribution^26^,^28^) is sister to subgenera *Euphorbia* (most diverse species of the genus distributed across tropics and subtropics^27^) and *Chamaesyce* (diverse growth forms including many New World species^26^) (Supplementary Figure 1). With regard to relationships below the subgenus level, despite relatively low resolution as would be expected due to the use of only one marker (*ndhF*), no well supported (PP > 0.95) incongruences with previous studies were found^26^,^27^,^28^,^29^.

We then investigated the phylogenetic distribution of medicinal plants on the phylogeny of *Euphorbia*. We found that plants used medicinally in the genus are significantly phylogenetically clustered (median = 0.76, p(D < 1): ***, Table 1). The EBDCS level 2 state categories of *medicines* that showed weak to moderate phylogenetic signal included *genitourinary system disorders* and *unspecified medicinal disorders* (Table 2).

**Figure 2:**
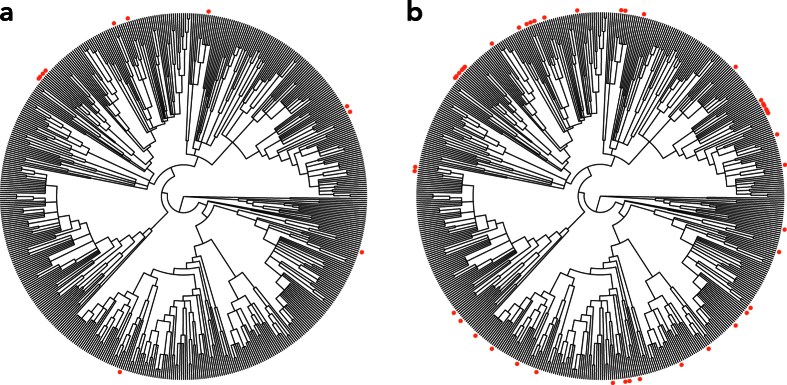
Phylogenetic distribution of species for (**a**) the Economic Botany Data Collection Standard (EBDCS) category *inflammation* and (**b**) the category *inflammatory response*. Red dots indicate species with documented use described in the category.

**Figure 3:**
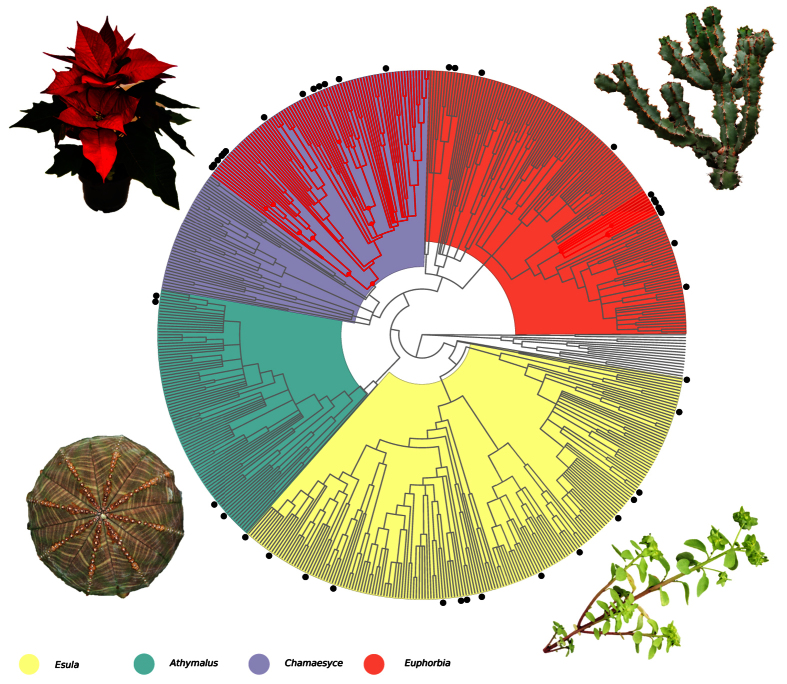
Hot nodes and corresponding clades of the category *inflammatory response*. Hot nodes (red dots) were identified by the *nodesigl* command in PHYLOCOM v4.2 on the majority consensus tree. Hot nodes indicate that the observed number of species in the category in that node is higher than expected by chance. Black dots indicate species with documented uses in the category *inflammatory response*. Photo: Mogens Trolle and Madeleine Ernst (*Euphorbia pulcherrima*).

Of all EBDCS categories, the category *inflammation* is the most comparable to our interpretation of plant uses modulating the inflammatory response. However, in contrast to the EBDCS category *inflammation*, our category *inflammatory response* comprises not only plant species potentially causing an anti- but also a pro-inflammatory effect. In the interest of investigating differences resulting from the different classifications of medicinal plant uses, we subsequently compared the two inflammation-related categories. The category *inflammatory response* included 44 species, four times as many species as the EBDCS category *inflammation* (11 species; Fig. 2). There were also differences in the phylogenetic distribution of plants used for these two categories: A more than two-fold increase of the phylogenetic diversity index (PD) from the EBDCS category *inflammation* (median = 5.70, 7.40%; range = 4.71–6.79, 6.32–8.60%) to the category *inflammatory response* (median = 14.08, 18.36%; range = 11.96–16.58, 16.78–20.03%) was observed. Given the differences in the groups of plant species identified by the different inflammation-related categories, we further investigated whether plant species from these two categories come from the same lineages. We estimated the phylogenetic similarity between these two plant groups using the MNTD metric. The category *inflammatory response* showed no significant similarity to the EBDCS category *inflammation* (median = 0.40, p-value: ns, Table 3). It also showed no significant phylogenetic overlap with any of the other EBDCS level 2 state medicinal categories (Table 3). However, neither the EBDCS *inflammation* category, nor the *inflammatory response* category showed significant phylogenetic clustering (median = 0.54 and 0.88, p(D < 1): ns, Table 2). As a tentative approach to narrow down the number of species selected for bioactivity screening within the category *inflammatory response*, we identified nodes that are significantly overrepresented by species in this category (hot nodes^8^; Fig. 3). Hot nodes were mainly found within subgenera *Chamaesyce* and *Euphorbia*.

## Discussion

Ethnomedicinal uses have inspired the discovery of many drugs in the past^2^,^15^,^38^. In recent decades, following the assumption that plant-derived chemicals are constrained to certain evolutionary plant lineages^4^,^5^,^6^, it was shown that plants used in ethnomedicine are not randomly distributed across taxonomic groups e.g.^5^,^6^,^39^. Taxonomic relationships were seen, therefore, as a way of predicting the occurrence and nature of useful chemicals in plants^5^,^6^. More recent studies have built upon this idea and with increasingly robust molecular phylogenetic methods at hand have added to the taxonomy-oriented approaches to informed drug discovery^7^,^8^,^9^,^10^,^11^,^12^,^13^,^14^.

In this study, we explored evolutionary patterns of medicinal properties in *Euphorbia*. Based on the mechanism of action of ingenol mebutate, we focused the investigation on described plant uses that possibly modulate an inflammatory response.

First, we looked at the phylogenetic distribution of medicinal plants on the phylogeny of *Euphorbia*. In agreement with previous studies on medicinal properties in other angiosperm lineages^8^,^9^,^11^,^12^,^14^, we found that *Euphorbia* species used medicinally are significantly phylogenetically clustered (Table 1). The EBDCS level 2 state categories of *medicines* that showed weak to moderate phylogenetic signal included *genitourinary system disorders* and *unspecified medicinal disorders* (Table 2). Biological activities specific to systems of the body (such as the genitourinary system) can be of highly diverse nature, therefore assumptions on distinct biological actions or chemical underpinnings of the signal cannot be made. In contrast, neither the EBDCS *inflammation* category, nor the *inflammatory response* category showed significant phylogenetic clustering. Our rationale in this study was that classification of medicinal plant use based on the biological response to the treatment can uncover phylogenetic patterns that reflect more accurately the presence of certain chemical compounds. Our findings suggest that inflammatory modulators are found in several *Euphorbia* lineages. This was recovered based on the EBDCS classification, but was even more pronounced by interpreting medicinal plant uses from a perspective of a biological response. There are several potential explanations for this pattern. First, plant chemicals can be homoplasious, occurring across distantly related lineages^40^. This may be due to convergent evolution, recycling of chemicals from the environment by the plant e.g.^41^ or production of chemicals by endophytic fungi, as well as environmental and ecological effects on plant chemistry^10^,^40^,^42^. Although some structural types of *Euphorbia* diterpenoids, to which also ingenol mebutate belongs, and which most likely exhibit pro-inflammatory properties^36^, have been described as taxonomically significant for the genus^43^, their distribution across the phylogeny of *Euphorbia* may still be random, since their production may be more strongly influenced by some of the above named factors, or is not significantly related to the evolutionary history of the genus. Conservation of gene clusters associated with diterpenoid biosynthesis was reported at a high taxonomic level of the plant family Euphorbiaceae^44^. This suggests that the ability of diterpenoid production is present throughout the genus *Euphorbia*^44^. However, especially some members of *Euphorbia* subg. *Chamaesyce* have been reported as not containing *Euphorbia* specific diterpenoids^34^, potentially having the responsible genes silenced according to ecological needs^40^. Second, bioactivity might not be related to any phylogenetic patterns, since different chemical compounds from different biosynthetic routes may show the same or similar bioactivities^45^. For example, structurally different chemicals derived from unrelated natural sources have shown inhibition of the tumour necrosis factor-α, a pro-inflammatory cytokine, which regulates inflammation and related disorders^45^. Inflammatory modulators targeted by the *inflammatory response* category can be of different chemical structural classes and might therefore not follow a distribution associated with the phylogeny of the genus. It is therefore plausible that the category *inflammatory response* comprises a broad spectrum of different chemical structures, which are not associated to the phylogenetic relationships of the plants producing them. Third, there is inherent bias in the collection of data on medicinal use, as it is a subjective process with several restrictions, such as underrepresentation of certain languages and access to literature not available online. Only a large-scale chemical exploration of *Euphorbia* species would be able to tease apart the underlying causes of the phylogenetic pattern observed here.

When comparing the EBDCS category *inflammation* to the *inflammatory response* category we observed a more than two-fold increase of the phylogenetic diversity index (PD). Not only were more species included in the category *inflammatory response*, but also their distribution over the phylogeny was wider. Furthermore, the category *inflammatory response* showed no significant similarity to the EBDCS category *inflammation,* according to the MNTD metric. It also showed no significant phylogenetic overlap with any of the other EBDCS level 2 state medicinal categories. Our findings thus indicate that the category *inflammatory response* highlighted a different group of species, and may reflect unexplored medicinal potential not recovered by a standard method of classification such as the EBDCS. As a tentative approach to narrow down the number of species to be screened at an early stage of drug discovery within the category *inflammatory response*, we identified nodes that are significantly overrepresented by species in this category (hot nodes; Fig. 3). Hot nodes were mainly found within subgenera *Chamaesyce* and *Euphorbia*, which together comprise over half of the diversity in *Euphorbia* (about 1,200 species^24^). These two subgenera have not been as extensively studied chemically as subgenus *Esula* (the subject of 70% chemical studies to date^34^,^35^). Despite mentioned limitations, we were able to highlight specific lineages with a potential overrepresentation of chemical compounds modulating an inflammatory response in humans, and these highlighted lineages are poorly studied for their chemistry. Therefore, our approach shows potential for novel discoveries of pharmacologically relevant compounds from understudied plant lineages.

Although plant-derived chemicals play a relatively minor role in drug discovery in the pharmaceutical industry nowadays, due to reasons related to the relatively labor-, time- and cost-intensive work with naturally derived chemicals, fear of duplication, intellectual property concerns or biodiversity conservation issues^46^,^47^,^48^ plant-derived drugs continue to be part of the list of essential medicines for priority diseases published by the WHO (WHO Model List of Essential Medicines, April 2015). Given the current decrease of new drugs brought to the market^46^, and the huge potential of plant-derived chemicals in providing medicinally relevant bioactivity, there is undoubtedly scope for innovation in identifying drug candidates. Our approach highlights clusters of species in *Euphorbia* subgenera *Chamaesyce* and *Euphorbia*, which compared to the European subgenus *Esula*, have largely been chemically under-investigated. Out of a total of 91 *Euphorbia* species, which have been investigated for their chemistry and pharmacology^34^,^35^, there are only five species of *Euphorbia* subgenus *Chamaesyce* and 24 species of *Euphorbia* subgenus *Euphorbia* (based on recent taxonomic and molecular phylogenetic studies^24^,^26^,^27^,^28^,^29^,^30^,^49^). The remaining 62 species form part of the European *Euphorbia* subgenus *Esula*.

Future large-scale chemical and pharmacological investigations of previously untested species will be able to show if selection based on a classification system reflecting the biological response to the treatment efficiently results in improved hit rates. The classification of plant medicinal uses proposed here was associated to the inflammatory response. The inflammatory response plays an important role in the efficacy of the treatment of actinic keratosis by ingenol mebutate, a diterpenoid isolated from *Euphorbia peplus*^32^,^36^. Despite its release to the market, ingenol mebutate is not sourced efficiently from *Euphorbia peplus* and alternative sources such as synthetic or biosynthetic approaches are being investigated^33^,^44^. Little is known about the biosynthetic pathway of *Euphorbia* diterpenoids and a synthetic route for ingenol mebutate production is not feasible yet^33^,^44^. Given the low percentage and uneven subgeneric distribution of species of the genus *Euphorbia* investigated chemically, it is likely to find species with higher production of ingenol mebutate or compounds with similar or other medicinally relevant bioactivity profiles. Based on our findings, chemical characterization and investigation of biological activities related to the inflammatory response of selected *Euphorbia* species will follow, using the evolutionary approach and methodologies of classification proposed in this study.

## Methods

### Phylogenetic hypothesis

In this study, we produced a more densely sampled phylogenetic hypothesis of *Euphorbia* compiling DNA sequence data from the plastid marker *ndhF* from a series of studies focusing on subgenera of the genus^26^,^27^,^28^,^29^. Our matrix included sequences (1789 base pairs) of 560 *Euphorbia* species (>25% of the genus) representing all known subgeneric clades (Supplementary Table 1). To root the tree and allow comparison with previous studies^24^,^26^,^27^,^28^,^29^,^30^, exemplars of 15 related genera representing the remainder of the Euphorbiaceae family were included as outgroup. Species names and corresponding GenBank accessions are listed in Supplementary Table 2. We produced a Bayesian phylogenetic hypothesis using the GTR + I + G model. All comparative phylogenetic analyses described below were performed on a randomly selected subset of 1,000 trees within the 95% credible set.

### Medicinal uses of species of the genus *Euphorbia*

Information on uses of species of the genus *Euphorbia* was drawn from an extensive database^31^, including plants used medicinally as well as for other purposes; such as animal food, environmental uses, materials, (non-) vertebrate poisons and social uses. The database contains over 1,000 use records referring to 156 *Euphorbia* species, of which 92 (63%) were included in our phylogenetic tree. The use records from the database were coded according to the Economic Botany Data Collection Standard (EBDCS)^17^, recommended by the Biodiversity Information Standards TDWG (http://www.tdwg.org).

### Identifying *Euphorbia* uses modulating an inflammatory response

In the present study we argue that standard approaches to medicinal plant use classification used in previous studies are potentially misleading for predictive purposes in early drug discovery. We propose that, when studying medicinal plant uses in a phylogenetic context, a classification system that reflects the biological response to the treatment may reflect more accurately the cellular or molecular mechanism of the condition that a plant is used to treat and thus can reveal more successfully underlying biological activities and chemical properties. To illustrate this we used the EBDCS as an example. The level 2 states of the use category *medicines* described by the EBDCS^17^ are mostly linked to systems of the human body or symptoms of a disease, allowing only little insight into the disease’s cause or assumptions on a distinct biological activity or chemical nature of the medicinal plants. Here, we explore an alternative way of classifying diseases treated by *Euphorbia* species. We suggest that the proposed classification gives a better insight into how the plant’s chemicals might interfere in the disease process (pharmacological effect^37^) and thus is a better proxy for the plant’s biological activity than a classification based on systems of the body.

We focus on described plant uses that suggest to modulate an inflammatory response. The inflammatory response is a protective response, which eliminates offending agents of cell injury (e.g., microbes, toxins) and its consequences (e.g., necrotic cells and tissues). On the other hand, inflammatory reactions also underlie many pathologic conditions and they are thought to contribute to a variety of diseases such as metabolic, degenerative, or genetic disorders (e.g. type 2 diabetes, Alzheimer, cancer)^50^,^51^. Inflammatory modulators can thus interact at many different levels of the inflammatory cascade, including many different mechanisms of action. By targeting a biological response with many possible molecular mechanisms of action of the drug candidates we increase the possibility of discovering new chemical compounds in future early stage drug discovery screening approaches without being restricted to chemical compounds with very similar structures, which would most likely be targeted by aiming for more specific mechanisms of action. Within the genus *Euphorbia*, almost 70% of all chemical compounds described to date are new^34^. The chance of finding previously undiscovered compounds within *Euphorbia* is therefore considerably high. Also, an inflammatory response can be deducted relatively easily from ethnomedicinal descriptions, in comparison to more specific mode of actions. We were particularly interested in medicinal plant uses modulating the inflammatory response because inflammatory processes have shown relevance in the treatment of actinic keratosis by ingenol mebutate^32^. *In vitro* and *in vivo* studies of the molecular and cellular mode of action of the treatment showed that initial cell death is followed by a complex inflammatory response crucial for preventing tumor relapse and responsible for the high efficacy of the treatment^32^,^36^. Given that the inflammatory response, in particular, plays an important role in the efficacy of the treatment, we aimed to identify species of *Euphorbia*, suggesting the ability to modulate an inflammatory response in humans.

With special focus on whether the described treatment by the medicinal plant triggers an inflammatory response in humans, we investigated all records in the database of uses of *Euphorbia*. We looked for indications of the presence of a potential anti- or pro-inflammatory agent. We used the definitions of diseases given on the PubMed Health Diseases and Conditions Database^52^ and Dorland’s Illustrated Medical Dictionary^53^, which include either descriptions of diseases or their treatment. Use reports were thereafter classified into three categories: *inflammatory response* (treatment that can be related to an inflammatory response; 44 species), *no inflammatory response* (treatment that can not be related to an inflammatory response; 12 species), and *unknown* (description of medicinal use that contains insufficient information for classification; 23 species). Besides records describing medicinal uses of *Euphorbia*, we also included records of toxicity found in the EBDCS level 1 state category *vertebrate poisons*. Toxicity is separated from medicinality only by dosage and may therefore be a convincing indicator of bioactivity^54^. An overview of the use data interpreted with the EBDCS categories and the *inflammatory response* categories is shown in Supplementary Tables 3 and 4.

### Evolutionary patterns of medicinal properties in *Euphorbia*

Here, we explored whether our interpretation of medicinal plant use can reveal different phylogenetic patterns, compared to a standard approach of classification. We performed three different comparative phylogenetic analyses.

First, we investigated the strength in phylogenetic signal of the EBDCS categories and the *inflammatory response* category using the D statistic^55^, a measure of phylogenetic signal, implemented by the function *phylo.d* in the R package *caper*^56^. Two p-values are calculated for the D statistic, p(D < 1) indicating whether the D metric is significantly smaller than 1, meaning that the trait (species’ medicinal properties) is not randomly distributed over the phylogeny. The second p-value, p(D > 0) indicates whether the D metric is significantly greater than 0, meaning that the trait (species’ medicinal properties) has a significantly different distribution on the phylogeny from the standard Brownian model of evolution. The phylogenetic signal is considered strong if p(D < 1) < 0.05 and p(D > 0) > 0.05^55^. In our study, phylogenetic signal was considered significant if >95% of the 1,000 trees showed a p(D < 1) value < 0.05 and the signal was considered strong if >95% of the 1,000 trees showed a p(D > 0) value > 0.05.

Second, we compared the phylogenetic diversity captured by plant species identified by the two different classification methods. To evaluate the phylogenetic diversity (PD) of species identified by the *inflammatory response* category and the EBDCS categories, we calculated the PD index (measuring the total branch length spanned by the tree of species in a given category) proposed by Faith^57^, and implemented in the function *pd* in the R package *picante* v.1.6-2^58^. The PD index was expressed as absolute value as well as a percentage of the PD of the total phylogenetic tree. High PD percentage means that species included in this category are spread across the whole tree, while low percentage means that the species are found in only few, clustered parts of the tree.

Third, we investigated the overlap in the plant lineages identified by the two classification methods. To do that, we compared the phylogenetic similarity of the *inflammatory response* category and the EBDCS categories, by calculating the mean nearest taxon distance (MNTD)^59^ – a measure showing the phylogenetic proximity of species between two categories on the tree - using the *comdistnt* function in the R package *picante* v.1.6-2^58^. P-values for the MNTD were calculated by comparing the MNTD value between the *inflammatory response* category and an EBDCS category to 1,000 randomly generated categories of the same size within the species pool of the *inflammatory response* category and the EBDCS category *medicines*. The two categories were considered phylogenetically significantly similar if at least 95% of the 1,000 trees showed a p-value < 0.05.

Further, as tentative approach to narrow down the number of species chosen for an early stage drug discovery screening, we identified the position (nodes in phylogeny) of phylogenetic clustering for the *inflammatory response* category. We highlighted so-called “hot nodes” on the phylogeny, i.e. nodes that are significantly overrepresented by species in a given category^8^,^11^, using the *nodesigl* command in PHYLOCOM v4.2^59^.

All analyses we describe were performed with in-house scripts in the R environment Version 3.0.3 (http://www.R- project.org/), which are available in the Supplementary Information. Additional information on all method subsections is presented in Supplementary Methods. The alignment file as well as the set of 1,000 Bayesian trees used for analysis was deposited on dryad (http://dx.doi.org/10.5061/dryad.s2df3).

## Additional Information

**How to cite this article**: Ernst, M. *et al*. Evolutionary prediction of medicinal properties in the genus *Euphorbia* L. *Sci. Rep.* **6**, 30531; doi: 10.1038/srep30531 (2016).

## Supplementary Material

Supplementary Information

## Figures and Tables

**Figure 1:**
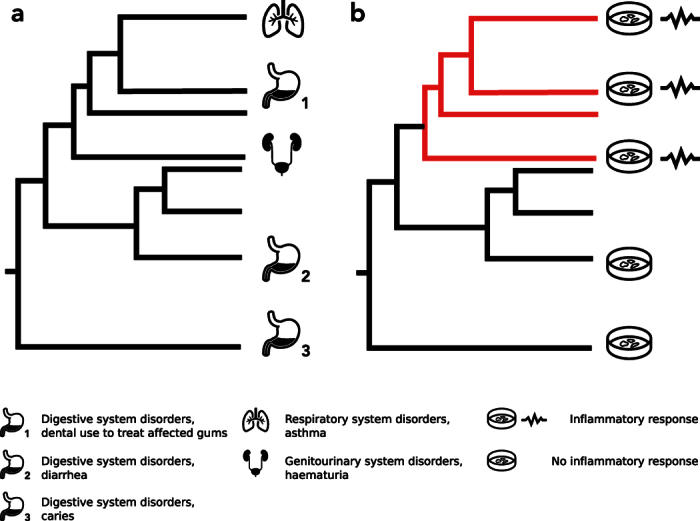
Hypothetical distribution of medicinal plant uses across a phylogeny. (**a)** Plant medicinal uses as classified by the Economic Botany Data Collection Standard based on systems of the body (**b)** Same plant medicinal uses classified based on a biological response. When seeking lineages with potential agents modulating an inflammatory response, the classification in (**a)** is not informative. Instead, the classification in (**b**) allows us to identify clades (marked in red in (**b)**) that are overrepresented in species potentially modulating an inflammatory response. Icons: thenounproject.com.

**Table 1:** Phylogenetic signal per EBDCS level 1 state categories.

	N	Prevalence	D-statistic		Phylogenetic signal	
			Median	Range	p(D < 1)^a^	strength^b^
Animal food	12	0.02	0.98	0.55–1.47	ns	weak
Environmental uses	18	0.03	0.90	0.56–1.27	ns	weak
Materials	15	0.03	0.88	0.61–1.21	ns	weak
**Medicines**	**65**	**0.12**	**0.76**	**0.65–0.88**	***	**weak**
Non-Vertebrate poisons	9	0.02	0.85	0.39–1.57	ns	weak
Social uses	8	0.01	0.82	0.15–1.51	ns	weak
Vertebrate poisons	46	0.08	1.06	0.90–1.26	ns	weak

Phylogenetic signal (D-statistic) on a randomly selected subset of 1,000 Bayesian trees within the 95% credible set of uses of *Euphorbia*^31^ classified into level 1 state categories according to the Economic Botany Data Collection Standard (EBDCS). Out of all level 1 state categories only *medicines* shows phylogenetic signal (in bold). N: Number of species.

^a^* 95% p < 0.05; *** all p < 0.005.

^b^weak: <90% p(D > 0) > 0.05; moderate: 90% p(D > 0) > 0.05; strong: 95% p(D > 0) > 0.05; very strong: all p(D > 0) > 0.05.

**Table 2:** Phylogenetic signal per EBDCS level 2 state categories *medicines* and the category *inflammatory response*.

	N	Prevalence	D-statistic		Phylogenetic signal	
			Median	Range	p(D < 1)^a^	strength^b^
Abnormalities	12	0.02	0.83	0.48–1.20	ns	weak
Digestive system disorders	26	0.05	0.78	0.51–1.04	ns	weak
**Genitourinary system disorders**	**9**	**0.02**	**0.35**	**−0.06–0.87**	*	**moderate**
Infections/infestations	21	0.04	0.82	0.50–1.15	ns	weak
Inflammation	11	0.02	0.54	0.09–1.27	ns	weak
Injuries	16	0.03	0.88	0.52–1.25	ns	weak
Pain	15	0.03	0.78	0.43–1.19	ns	weak
Respiratory system disorders	10	0.02	0.89	0.39–1.38	ns	weak
Skin-/subcutaneous cellular tissue disorders	27	0.05	0.73	0.50–0.95	ns	weak
**Unspecified medicinal disorders**	**27**	**0.05**	**0.65**	**0.43–0.88**	*	**weak**
Inflammatory response	44	0.08	0.88	0.68–1.03	ns	weak
No inflammatory response	12	0.02	0.85	0.49–1.29	ns	weak
Unknown	23	0.04	1.05	0.77–1.37	ns	weak

Phylogenetic signal (D-statistic) on a randomly selected subset of 1,000 Bayesian trees within the 95% credible set of uses of *Euphorbia*^31^ classified into level 2 state categories of *medicines* according to the Economic Botany Data Collection Standard (EBDCS) and the category *inflammatory response*. EBDCS categories *genitourinary system disorders* and *unspecified medicinal disorders* show phylogenetic signal (in bold), while the EBDCS category *inflammation* and the category *inflammatory response* don’t. N: Number of species.

^a^* 95% p < 0.05; *** all p < 0.005.

^b^weak: <90% p(D > 0) > 0.05; moderate: 90% p(D > 0) > 0.05; strong: 95% p(D > 0) > 0.05; very strong: all p(D > 0) > 0.05.

**Table 3:** Phylogenetic similarity between the category *inflammatory response* and the EBDCS level 2 state categories *medicines*.

	N	Prevalence	MNTD		
			Median	Range	p-value
Abnormalities	12	0.02	0.35	0.29–0.42	ns
Digestive system disorders	26	0.05	0.19	0.16–0.22	ns
Genitourinary system disorders	9	0.02	0.59	0.46–0.71	ns
Infections/infestations	21	0.04	0.14	0.12–0.18	ns
Inflammation	11	0.02	0.40	0.33–0.49	ns
Injuries	16	0.03	0.25	0.21–0.30	ns
Pain	15	0.03	0.29	0.24–0.34	ns
Respiratory system disorders	10	0.02	0.40	0.34–0.47	ns
Skin-/subcutaneous cellular tissue disorders	27	0.05	0.15	0.13–0.19	ns
Unspecified medicinal disorders	27	0.05	0.18	0.16–0.21	ns

Mean nearest taxon distance (MNTD) between species in the Economic Botany Data Collection Standard (EBDCS) level 2 state *medicines* categories and the category *inflammatory response*. The category *inflammatory response* does not show significant phylogenetic similarity with any of the EBDCS categories and thus is sufficiently sensitive to highlight a different group of species, eventually reflecting unexplored medicinal potential not recovered by the EBDCS.

## References

[b1] Newman D. J., Cragg G. M. & Snader K. M. The influence of natural products upon drug discovery. Nat. Prod. Rep. 17, 215–234 (2000). 10888010 10.1039/a902202c

[b2] Cragg G. M. & Newman D. J. Natural products: A continuing source of novel drug leads. Biochim. Biophys. Acta 1830, 3670–3695 (2013). 23428572 10.1016/j.bbagen.2013.02.008 PMC3672862

[b3] *Plants under pressure – a global assessment*. IUCN Sampled Red List Index for Plants (Royal Botanic Gardens, Kew, UK, 2012).

[b4] Dahlgren R. M. T. A revised system of classification of the angiosperms. Bot. J. Linn. Soc. 80, 91–124 (1980).

[b5] Gottlieb O. R. Ethnopharmacology versus chemosytematics in the search for biologically active principles in plants. J. Ethnopharmacol. 6, 227–238 (1982). 6127441 10.1016/0378-8741(82)90005-8

[b6] Gottlieb O. R., Borin M. R. de M. B. & De Brito N. R. S. Integration of ethnobotany and phytochemistry: dream or reality? Phytochemistry 60, 145–152 (2002). 12009317 10.1016/s0031-9422(02)00088-2

[b7] Rønsted N., Savolainen V., Mølgaard P. & Jäger A. K. Phylogenetic selection of *Narcissus* species for drug discovery. Biochem. Syst. Ecol. 36, 417–422 (2008).

[b8] Saslis-Lagoudakis C. H., Klitgaard B. B., Forest F., Francis L., Savolainen V., Williamson E. M. & Hawkins J. A. The use of phylogeny to interpret cross-cultural patterns in plant use and guide medicinal plant discovery: an example from *Pterocarpus* (Leguminosae). PLoS One 6, e22275 (2011). 21789247 10.1371/journal.pone.0022275 PMC3138776

[b9] Zhu F. . Clustered patterns of species origins of nature-derived drugs and clues for future prospecting. Proc. Natl. Acad. Sci. USA 108, 12943–12948 (2011). 21768386 10.1073/pnas.1107336108 PMC3150889

[b10] Rønsted N. . Can phylogeny predict chemical diversity and potential medicinal activity of plants? A case study of Amaryllidaceae. BMC Evol. Biol. 12, 182 (2012). 10.1186/1471-2148-12-182 PMC3499480 22978363

[b11] Saslis-Lagoudakis C. H. . Phylogenies reveal predictive power of traditional medicine in bioprospecting. Proc. Natl. Acad. Sci. USA 109, 15835–15840 (2012). 22984175 10.1073/pnas.1202242109 PMC3465383

[b12] Grace O. M. . Evolutionary history and leaf succulence as explanations for medicinal use in aloes and the global popularity of *Aloe vera*. BMC Evol. Biol. 15, 29 (2015). 10.1186/s12862-015-0291-7 PMC4342203 25879886

[b13] Tao L. . Clustered distribution of natural product leads of drugs in chemical space as influenced by the privileged target-sites. Sci. Rep. 5, 9325 (2015). 25790752 10.1038/srep09325 PMC5380136

[b14] Yessoufou K., Daru B. H. & Muasya A. M. Phylogenetic exploration of commonly used medicinal plants in South Africa. Mol. Ecol. Resour. 15, 405–413 (2015). 25066923 10.1111/1755-0998.12310

[b15] Fabricant D. S. & Farnsworth N. R. The value of plants used in traditional medicine for drug discovery. Environ. Health Perspect 109, 69–75 (2001). 11250806 10.1289/ehp.01109s169 PMC1240543

[b16] Heinrich M., Edwards S., Moerman D. E. & Leonti M. Ethnopharmacological field studies: A critical assessment of their conceptual basis and methods. J. Ethnopharmacol. 124, 1–17 (2009). 19537298 10.1016/j.jep.2009.03.043

[b17] Cook F. E. M. Economic Botany Data Collection Standard (Royal Botanic Gardens, Kew, UK, 1995).

[b18] Gruca M., Cámara-Leret R., Macía M. J. & Balslev H. New categories for traditional medicine in the Economic Botany Data Collection Standard. J. Ethnopharmacol. 155, 1388–1392 (2014). 24971798 10.1016/j.jep.2014.06.047

[b19] Oritz de Montellano B. Empirical Aztec medicine. Science 188, 215–220 (1975). 1090996 10.1126/science.1090996

[b20] Staub O. P., Geck M. S., Weckerle C. S., Casu L. & Leonti M. Classifying diseases and remedies in ethnomedicine and ethnopharmacology. J. Ethnopharmacol. 174, 514–519 (2015). 26342522 10.1016/j.jep.2015.08.051

[b21] Snider G. L. Nosology for our day: Its application to chronic obstructive pulmonary disease. Am. J. Respir. Crit. Care Med. 167, 678–683 (2003). 12598211 10.1164/rccm.200203-204PP

[b22] Janssens A. C. J. W. & Van Duijn M. Genome-based prediction of common diseases: advances and prospects. Human Molecular Genetics 17, R166–R173 (2008). 18852206 10.1093/hmg/ddn250

[b23] Govaerts R., Frodin D. G. & Radcliffe-Smith A. World checklist and bibliography of Euphorbiaceae (with Pandaceae). Vol. 2 (Royal Botanic Gardens, Kew, UK, 2000).

[b24] Horn J. W. . Phylogenetics and the evolution of major structural characters in the giant genus *Euphorbia* L. (Euphorbiaceae). Mol. Phylogenet. Evol. 63, 305–326 (2012). 22273597 10.1016/j.ympev.2011.12.022

[b25] Frodin D. G. History and concepts of big plant genera. TAXON 53, 753–776 (2004).

[b26] Yang Y. . Molecular phylogenetics and classification of *Euphorbia* subgenus *Chamaesyce* (Euphorbiaceae). TAXON 61, 764–789 (2012).

[b27] Dorsey B. L. . Phylogenetics, morphological evolution, and classification of *Euphorbia* subgenus *Euphorbia* (Euphorbiaceae). TAXON 62, 291–315 (2013).

[b28] Peirson J. A., Bruyns P. V., Riina R., Morawetz J. J. & Berry P. E. A molecular phylogeny and classification of the largely succulent and mainly African *Euphorbia* subg. *Athymalus* (Euphorbiaceae). TAXON 62, 1178–1199 (2013).

[b29] Riina R. . A worldwide molecular phylogeny and classification of the leafy spurges, *Euphorbia* subgenus *Esula* (Euphorbiaceae). TAXON 62, 316–342 (2013).

[b30] Horn J. W. . Evolutionary bursts in *Euphorbia* (Euphorbiaceae) are linked with photosynthetic pathway. Evolution 68, 3485–3504 (2014). 25302554 10.1111/evo.12534

[b31] Ernst M. . Global medicinal uses of *Euphorbia* L. (Euphorbiaceae). J. Ethnopharmacol. 176, 90–101 (2015). 26485050 10.1016/j.jep.2015.10.025

[b32] Berman B. New developments in the treatment of actinic keratosis: focus on ingenol mebutate gel. Clin. Cosmet. Investig. Dermatol. 20, 111–122 (2012). 10.2147/CCID.S28905 PMC3430094 22956883

[b33] Jørgensen L. . 14-step synthesis of (+)-ingenol from (+)-3-carene. Science 341, 878–882 (2013). 23907534 10.1126/science.1241606

[b34] Vasas A. & Hohmann J. *Euphorbia* diterpenes: isolation, structure, biological activity, and synthesis (2008–2012). Chem. Rev. 114, 8579–8612 (2014). 25036812 10.1021/cr400541j

[b35] Shi Q. W., Su X. H. & Kiyota H. Chemical and pharmacological research of the plants in genus *Euphorbia*. Chem. Rev. 108, 4295–4327 (2008). 18817355 10.1021/cr078350s

[b36] Kedei N. . Characterization of the interaction of ingenol 3-angelate with Protein Kinase C. Cancer Res. 64, 3243–3255 (2004). 15126366 10.1158/0008-5472.can-03-3403

[b37] Vallance P. & Smart T. G. The future of pharmacology. Br. J. Pharmacol. 147, S304–S307 (2006). 16402118 10.1038/sj.bjp.0706454 PMC1760753

[b38] Balandrin M. F., Kinghorn A. D. & Farnsworth N. R. Plant-derived natural products in drug discovery and development. In Human Medicinal Agents from plant s, Vol. 534 (ed. Kinghorn D. & Balandrin M. F.) Ch. 1, 2–12 (American Chemical Society, 10.1021/bk-1993-0534.ch001, 1993).

[b39] Moerman D. E. The medicinal flora of native North America: An analysis. J. Ethnopharmacol. 31, 1–42 (1991). 2030588 10.1016/0378-8741(91)90141-y

[b40] Wink M., Botschen F., Gosmann C., Schäfer H. & Waterman P. G. Chemotaxonomy seen from a phylogenetic perspective and evolution of secondary metabolism. In Biochemistry of plant secondary metabolism Edn. 2 Annual Plant Reviews Vol. 40, 364–33 (ed. Wink M.) (Wiley-Blackwell, Oxford, UK, 2010).

[b41] Kusari S. . Tramadol – A true natural product? Angew. Chem. Int. Ed. 53, 1–5 (2014). 10.1002/anie.201406639 25219922

[b42] Ernst M. . A metabolomics protocol for plant systematics by matrix-assisted laser-desorption/ionization time-of flight mass spectrometry. Anal. Chim. Acta 859, 46–58 (2015). 25622605 10.1016/j.aca.2015.01.002

[b43] Evans F. J. & Kinghorn A. D. A comparative phytochemical study of the diterpenes of some species of the genera *Euphorbia* and *Elaeophorbia* (Euphorbiaceae). Bot. J. Linn. Soc. 74, 23–35 (1977).

[b44] King A. J., Brown G. D., Gilday A. D., Larson T. R. & Graham I. A. Production of bioactive diterpenoids in the Euphorbiaceae depends on evolutionary conserved gene clusters. Plant Cell 26, 3286–3298 (2014). 25172144 10.1105/tpc.114.129668 PMC4371829

[b45] Paul A. T., Gohil V. M. & Bhutani K. K. Modulating TNF-α signalling with natural products. Drug Discov. Today 11, 725–732 (2006). 16846800 10.1016/j.drudis.2006.06.002

[b46] Li J. W. H. & Vederas J. C. Drug discovery and natural products: End of an era or an endless frontier? Science 325, 161–165 (2009). 19589993 10.1126/science.1168243

[b47] Kingston D. G. I. Modern natural products drug discovery and its relevance to biodiversity conservation. J. Nat. Prod. 74, 496–511 (2011). 21138324 10.1021/np100550t PMC3061248

[b48] Scannell J. W., Blanckley A., Boldon H. & Warrington B. Diagnosing the decline in pharmaceutical R&D efficiency. Nat. Rev. Drug Discov. 11, 191–200 (2012). 22378269 10.1038/nrd3681

[b49] Riina R. & Berry P. E. (coordinators), *Euphorbia planetary biodiversity inventory database*. (2012) Available at: http://app.tolkin.org/projects/72/taxa. (Accessed: 29th April 2015).

[b50] Medzhitov R. Origin and physiological roles of inflammation. Nature 454, 428–435 (2008). 18650913 10.1038/nature07201

[b51] Kumar V., Abbas A. K. & Aster J. C. Robbins and Cotran pathologic basis of disease 9^th^ edn, (ed. Kumar V., Abbas A. K. & Aster J. C.) Ch. 3, 69–112 (Elsevier, 2015).

[b52] *A.D.A.M. Medical Encyclopedia*. (1997-2011) Available at: http://www.ncbi.nlm.nih.gov/pubmedhealth/s/diseases_and_conditions/. (Accessed: 29th April 2015).

[b53] Dorland W. A. N. Dorland’s illustrated medical dictionary Edn. 32 (Elsevier, Philadelphia, USA, 2012).

[b54] Stumpf W. E. The dose makes the medicine. Drug Discov. Today 11, 550–555 (2006). 16713907 10.1016/j.drudis.2006.04.012

[b55] Fritz S. A. & Purvis A. Selectivity in mammalian extinction risk and threat types: A new measure of phylogenetic signal strength in binary traits. Conserv. Biol. 24, 1042–1051 (2010). 20184650 10.1111/j.1523-1739.2010.01455.x

[b56] Orme D. . *caper: Comparative analyses of phylogenetics and evolution in R*. R package version 0.5.2. (2013) Available at: http://CRAN.R-project.org/package=caper. (Accessed: 15th January 2016).

[b57] Faith D. P. Conservation evaluation and phylogenetic diversity. Biol. Cons. 61, 1–10 (1992).

[b58] Kembel S. W. Picante: R tools for integrating phylogenies and ecology. Bioinformatics 26, 1463–1464 (2010). 20395285 10.1093/bioinformatics/btq166

[b59] Webb C. O., Ackerly D. D. & Kembel S. W. Phylocom: software for the analysis of phylogenetic community structure and trait evolution. Bioinformatics 24, 2098–2100 (2008). 18678590 10.1093/bioinformatics/btn358

