# Supplementary material for: Evolutionary prediction of medicinal properties in the genus Euphorbia L
Source: Sci Rep. 2016 Jul 28;6:30531. doi: 10.1038/srep30531 (PMC4964329; doi:10.1038/srep30531)
Supplement: Supplementary Information [file srep30531-s1.pdf]

Supplementary Information

Evolutionary prediction of medicinal properties in the genus *Euphorbia* L.

Madeleine Ernst[1], C. Haris Saslis-Lagoudakis[1], Olwen M. Grace[2], Niclas Nilsson[3], Henrik Toft Simonsen[4], James W. Horn[5], Nina Rønsted[1]

[1] Natural History Museum of Denmark, Faculty of Science, University of Copenhagen, Sølvgade 83S, DK-1307 Copenhagen K, Denmark
[2] Comparative Plant & Fungal Biology, Royal Botanic Gardens, Kew, Surrey TW9 3AB, United Kingdom
[3] Skin Research, LEO Pharma A/S, Industriparken 55, DK-2750 Ballerup, Denmark
[4] Department of Systems Biology, Technical University of Denmark, DK-2800 Kgs. Lyngby, Denmark
[5] Natural and Applied Sciences, University of Wisconsin, Green Bay, LS 458, UW-Green Bay, 2420 Nicolet Dr, Green Bay, WI 54311-7001, USA

Supplementary Methods

Phylogenetic hypothesis. Although previous studies have used various DNA markers, we found that the plastid marker *ndhF*, as well as the nuclear *ITS* were the ones that have been extensively sampled across subgenera. However, high divergence of *ITS* sequences within *Euphorbia* created a high level of alignment ambiguity at the genus level. Therefore, we based our phylogenetic analysis only on the *ndhF* sequence data. Sequences were aligned using MAFFT Version 7.205 [1] with default parameters and subsequent manual adjustments were made in Mesquite Version 3.01 [2]. Indels were in triplets apart from autapomorphic indels in certain sequences and in non-protein coding parts of the sequence. The nucleotide substitution model was determined in jModelTest version 2.1.7 [3,4]. Using the Akaike Information Criterion[5] (AIC) GTR + I + G was selected as best model. Bayesian MCMC analyses[6] were performed using MrBayes version 3.2.5 [7]. Two concurrent runs of 4 chains of 1 x 10^7 generations were executed, sampling every 500 generations. Examination of the trace files in Tracer 1.6.0 [8] demonstrated convergence of the runs and the average standard deviation of split frequencies in MrBayes reached a value below 0.007. Tracer was also used to ensure an effective sample size of > 200 for all parameters. The plot of the log likelihood values in Tracer indicated a relative burn-in period of 10%. Therefore, the first 10% of the trees from each run were disregarded when computing the consensus tree in MrBayes.

Medicinal uses of species of the genus *Euphorbia*. In all phylogenetic manipulations, information below the species rank was not taken into account (all relevant information was merged at the species rank). Species in the phylogenetic tree with no record in the database[9] were scored as having no use (assigning 0 as the binary trait in the data matrix).

Identifying *Euphorbia* uses modulating an inflammatory response. A use record in our database[9] was allocated to the *inflammatory response* category if either present treatment methods could likely mediate an inflammatory response or if the symptom of the disease for which the plant species is used could be associated to an inflammatory response. Species were assigned to the *inflammatory response* category, when at least one of their described uses in the database[9] could be linked to an inflammatory response.

Evolutionary patterns of medicinal properties in *Euphorbia*. The D statistic is independent of tree size and trait prevalence (number of species in one category divided by the total number of species in the tree) if the tree has at least 50 terminals[10]. Our tree of 560 species therefore allowed the assumption of an unbiased estimate for D. However, cases of extremely low prevalence (e.g. 0.02 and under, or up to 8 species in a category in our study) have not been tested by Fritz and Purvis[10]. For our study, we considered categories with prevalence below 0.02 as potentially biased and therefore did not include them in any analysis. All comparative analyses were performed on phylograms with branch lengths proportional to DNA changes. Trees were only made ultrametric using the *chronopl* function in the R package *ape*[11] after statistical analyses for visualization purposes. Additional to the R packages mentioned in the main text also *geiger*[12] and *plyr*[13] were used. For graphical representation the tree drawing tool FigTree Version 1.4.2 (http://tree.bio.ed.ac.uk/software/figtree/) as well as the R environment extended by corresponding packages were used.

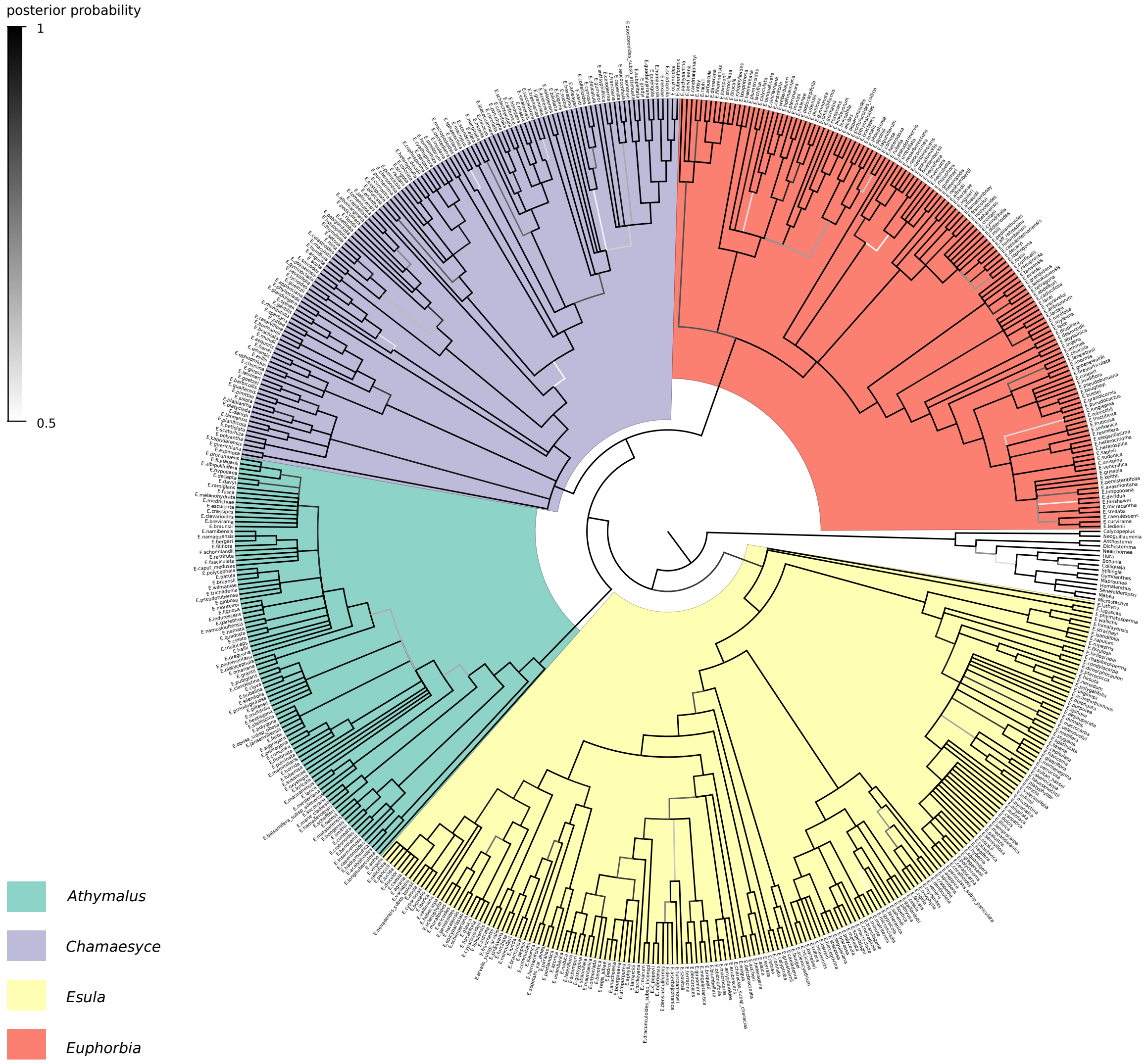

Supplementary Figure 1. Bayesian majority consensus tree. The tree is based on publicly available sequences of the marker *ndhF*. Branches are coloured according to the posterior probabilities.

Supplementary Table 1. Species sampled per subgeneric clade. Number of (medicinal) species sampled and corresponding estimated number of species per subgeneric clade, according to[14–17].

Clade	Sampled	Estimated	Medicinal species
Athymalus	91 (60%)	150	8
Chamaesyce	127 (21%)	600	19
Esula	190 (40%)	480	23
Euphorbia	137 (21%)	>650	15
**Total**	**560 (30%)**	**>1880**	**65**

Supplementary Table 2. GenBank accession numbers. Names and GenBank accession numbers of species sampled.

	Genus	Species	Author		GenBank	Clade
1	*Euphorbia*	*acalyphoides*	Hochst. ex Boiss.		AF538251	*Athymalus*
2	*Euphorbia*	*aggregata*	A. Berger		JN249097	*Athymalus*
3	*Euphorbia*	*albipollinifera*	L.C. Leach		KF267330	*Athymalus*
4	*Euphorbia*	*antso*	Denis		JN249102	*Athymalus*
5	*Euphorbia*	*balsamifera*		subsp. *adenensis* (Deflers) P. R. O. Bally	KF267335	*Athymalus*
6	*Euphorbia*	*benthamii*	Hiern		KF267336	*Athymalus*
7	*Euphorbia*	*bergeri*	N. E. Br.		JN249109	*Athymalus*
8	*Euphorbia*	*bongensis*	Kotschy and Peyr. ex Boiss.		KC019860	*Athymalus*
9	*Euphorbia*	*braunsii*	N. E. Br.		KF267341	*Athymalus*
10	*Euphorbia*	*brevirama*	N. E. Br.		KF267342	*Athymalus*
11	*Euphorbia*	*bruynsii*	L.C. Leach		KF267343	*Athymalus*
12	*Euphorbia*	*bubalina*	Boiss.		JN249112	*Athymalus*
13	*Euphorbia*	*caperonioides*	R. A. Dyer and P. G. Mey.		KF267346	*Athymalus*
14	*Euphorbia*	*caput-medusae*	L.		KF267347	*Athymalus*
15	*Euphorbia*	*celata*	R. A. Dyer		KF267350	*Athymalus*
16	*Euphorbia*	*clandestina*	Jacq.		KF267351	*Athymalus*
17	*Euphorbia*	*clava*	Jacq.		JN249117	*Athymalus*
18	*Euphorbia*	*clavarioides*	Boiss.		KF267352	*Athymalus*
19	*Euphorbia*	*crassipes*	Marloth		KF267355	*Athymalus*
20	*Euphorbia*	*crotonoides*	Boiss.		JQ750784	*Athymalus*
21	*Euphorbia*	*cumulata*	R. A. Dyer		KF267358	*Athymalus*
22	*Euphorbia*	*cuneata*	Vahl		KF267359	*Athymalus*
23	*Euphorbia*	*davyi*	N. E. Br.		KF267361	*Athymalus*
24	*Euphorbia*	*decepta*	N. E. Br.		KF267365	*Athymalus*
25	*Euphorbia*	*dregeana*	E. Mey. ex Boiss.		JN249130	*Athymalus*
26	*Euphorbia*	*esculenta*	Marloth		KF267366	*Athymalus*
27	*Euphorbia*	*fasciculata*	Thunb.		JN249137	*Athymalus*

Supplementary Table 2 continued. **GenBank accession numbers**. Names and GenBank accession numbers of species sampled.

	Genus	Species	Author	GenBank	Clade
28	*Euphorbia*	*ferox*	Marloth	KF267368	*Athymalus*
29	*Euphorbia*	*filiflora*	Marloth	JN249139	*Athymalus*
30	*Euphorbia*	*fimbriata*	Scop.	JN249140	*Athymalus*
31	*Euphorbia*	*flanaganii*	N. E. Br.	JN249141	*Athymalus*
32	*Euphorbia*	*friedrichiae*	Dinter	KF267373	*Athymalus*
33	*Euphorbia*	*fusca*	Marloth	JN249145	*Athymalus*
34	*Euphorbia*	*gariepina*	Boiss.	JN249146	*Athymalus*
35	*Euphorbia*	*globosa*	(Haw.) Sims	JN249149	*Athymalus*
36	*Euphorbia*	*grantii*	Oliv.	JN249153	*Athymalus*
37	*Euphorbia*	*hadramautica*	Baker	KC019792	*Athymalus*
38	*Euphorbia*	*hallii*	R. A. Dyer	KF267376	*Athymalus*
39	*Euphorbia*	*hamaderoensis*	A. G. Mill.	KF267377	*Athymalus*
40	*Euphorbia*	*hamata*	(Haw.) Sweet	AF538237	*Athymalus*
41	*Euphorbia*	*heptagona*	L.	KF267378	*Athymalus*
42	*Euphorbia*	*horrida*	Boiss.	JN249162	*Athymalus*
43	*Euphorbia*	*hypogaea*	Marloth	KF267381	*Athymalus*
44	*Euphorbia*	*indurescens*	L.C. Leach	KF267382	*Athymalus*
45	*Euphorbia*	*insarmentosa*	P. G. Mey.	KF267383	*Athymalus*
46	*Euphorbia*	*jansenvillensis*	Nel	JN249166	*Athymalus*
47	*Euphorbia*	*larica*	Boiss.	KC212430	*Athymalus*
48	*Euphorbia*	*lignosa*	Marloth	JN249173	*Athymalus*
49	*Euphorbia*	*longituberculosa*	Hochst. ex Boiss.	AF538252	*Athymalus*
50	*Euphorbia*	*loricata*	Lam.	KF267395	*Athymalus*
51	*Euphorbia*	*mammillaris*	L.	KF267400	*Athymalus*
52	*Euphorbia*	*marie-cladieae*	Rzepecky	KF267401	*Athymalus*
53	*Euphorbia*	*masirahensis*	Ghaz.	KF267402	*Athymalus*
54	*Euphorbia*	*matabelensis*	Pax	KF267403	*Athymalus*
55	*Euphorbia*	*melanohydrata*	Nel	KF267404	*Athymalus*

Supplementary Table 2 continued. **GenBank accession numbers**. Names and GenBank accession numbers of species sampled.

	Genus	Species	Author		GenBank	Clade
56	*Euphorbia*	*meuleniana*	O. Schwartz		KF267405	*Athymalus*
57	*Euphorbia*	*monteiroi*	Hook.		KF267406	*Athymalus*
58	*Euphorbia*	*multiceps*	A. Berger		KF267408	*Athymalus*
59	*Euphorbia*	*multifolia*	A. C. White		KF267409	*Athymalus*
60	*Euphorbia*	*namaquensis*	N. E. Br.		KF267410	*Athymalus*
61	*Euphorbia*	*namibensis*	Marloth		KF267411	*Athymalus*
62	*Euphorbia*	*namuskluftensis*	L. C. Leach		JN249182	*Athymalus*
63	*Euphorbia*	*oatesii*	Rolfe		KF267412	*Athymalus*
64	*Euphorbia*	*obesa*		subsp. *obesa* Hook. f.	JN249189	*Athymalus*
65	*Euphorbia*	*omariana*	M. G. Gilbert		AF538243	*Athymalus*
66	*Euphorbia*	*oxystegia*	Boiss.		KF267413	*Athymalus*
67	*Euphorbia*	*patula*	Mill.		KF267414	*Athymalus*
68	*Euphorbia*	*pedemontana*	L.C. Leach		JN249195	*Athymalus*
69	*Euphorbia*	*pentagona*	Haw.		KF267415	*Athymalus*
70	*Euphorbia*	*pillansii*	N. E. Br.		KF267416	*Athymalus*
71	*Euphorbia*	*platycephala*	Pax		AF538241	*Athymalus*
72	*Euphorbia*	*polycephala*	Marloth		JN249202	*Athymalus*
73	*Euphorbia*	*polygona*	Haw.		KF267292	*Athymalus*
74	*Euphorbia*	*procumbens*	Mill.		KF267420	*Athymalus*
75	*Euphorbia*	*pseudoglobosa*	Marloth		JN249205	*Athymalus*
76	*Euphorbia*	*pseudotuberosa*	Pax		KF267423	*Athymalus*
77	*Euphorbia*	*pubiglans*	N. E. Br.		KF267424	*Athymalus*
78	*Euphorbia*	*pulvinata*	Marloth		KF267426	*Athymalus*
79	*Euphorbia*	*quadrata*	Nel		KF267427	*Athymalus*
80	*Euphorbia*	*ramiglans*	N. E. Br.		JN249211	*Athymalus*
81	*Euphorbia*	*restituta*	N. E. Br.		KF267428	*Athymalus*
82	*Euphorbia*	*scheffleri*	Pax		JN249220	*Athymalus*
83	*Euphorbia*	*schoenlandii*	Pax		JN249222	*Athymalus*

Supplementary Table 2 continued. **GenBank accession numbers**. Names and GenBank accession numbers of species sampled.

	Genus	Species	Author	GenBank	Clade
84	*Euphorbia*	*silenifolia*	(Haw.) Sweet	KF267430	*Athymalus*
85	*Euphorbia*	*smithii*	S. Carter	JN249227	*Athymalus*
86	*Euphorbia*	*socotrana*	Balf. f.	JN249228	*Athymalus*
87	*Euphorbia*	*stellispina*	Haw.	KF267431	*Athymalus*
88	*Euphorbia*	*susannae*	Marloth	KF267432	*Athymalus*
89	*Euphorbia*	*trichadenia*	Pax	AF538236	*Athymalus*
90	*Euphorbia*	*tuberosa*	L.	AF538246	*Athymalus*
91	*Euphorbia*	*wilmaniea*	Marloth	KF267434	*Athymalus*
92	*Euphorbia*	*acerensis*	Boiss.	JQ750757	*Chamaesyce*
93	*Euphorbia*	*acuta*	Engelm.	AF538176	*Chamaesyce*
94	*Euphorbia*	*aequoris*	N. E. Br.	JQ750759	*Chamaesyce*
95	*Euphorbia*	*alata*	Hook.	JQ750760	*Chamaesyce*
96	*Euphorbia*	*albomarginata*	Torr. and A. Gray	JQ750762	*Chamaesyce*
97	*Euphorbia*	*angusta*	Engelm.	JQ750763	*Chamaesyce*
98	*Euphorbia*	*antisyphilitica*	Zucc.	JQ750764	*Chamaesyce*
99	*Euphorbia*	*appariciana*	Rizzini	JQ750765	*Chamaesyce*
100	*Euphorbia*	*arteagae*	W. R. Buck and Huft	JQ750766	*Chamaesyce*
101	*Euphorbia*	*barbicollis*	P. R. O. Bally	JQ750767	*Chamaesyce*
102	*Euphorbia*	*bifurcata*	Engelm.	AF538173	*Chamaesyce*
103	*Euphorbia*	*bilobata*	Engelm.	JQ750769	*Chamaesyce*
104	*Euphorbia*	*brachiata*	(E. Mey. ex Klotzsch and Garcke) Boiss.	JQ750770	*Chamaesyce*
105	*Euphorbia*	*burmanni*	(Klotzsch and Garcke) E. Mey. ex Boiss.	JQ750772	*Chamaesyce*
106	*Euphorbia*	*californica*	Benth.	JQ750773	*Chamaesyce*
107	*Euphorbia*	*caperata*	McVaugh	JQ750774	*Chamaesyce*
108	*Euphorbia*	*capitellata*	Engelm.	JQ750775	*Chamaesyce*
109	*Euphorbia*	*caterviflora*	N. E. Br.	JQ750776	*Chamaesyce*

Supplementary Table 2 continued. **GenBank accession numbers**. Names and GenBank accession numbers of species sampled.

	Genus	Species	Author		GenBank	Clade
110	*Euphorbia*	*celastroides*	Boiss.		JQ750777	*Chamaesyce*
111	*Euphorbia*	*ceroderma*	I. M. Johnst.		AF538153	*Chamaesyce*
112	*Euphorbia*	*chersina*	N. E. Br.		JQ750778	*Chamaesyce*
113	*Euphorbia*	*chersonesa*	Huft		AF538174	*Chamaesyce*
114	*Euphorbia*	*colletioides*	Benth.		JQ750779	*Chamaesyce*
115	*Euphorbia*	*colorata*	Engelm.		JQ750780	*Chamaesyce*
116	*Euphorbia*	*cornastra*	(Dressler) Radcl.-Sm.		JQ750781	*Chamaesyce*
117	*Euphorbia*	*corollata*	L.		JQ750782	*Chamaesyce*
118	*Euphorbia*	*cotinifolia*	L.		JQ750783	*Chamaesyce*
119	*Euphorbia*	*cuphosperma*	(Engelm.) Boiss.		JQ750785	*Chamaesyce*
120	*Euphorbia*	*cyathophora*	Murray		JQ750786	*Chamaesyce*
121	*Euphorbia*	*cymosa*	Poir.		JQ750787	*Chamaesyce*
122	*Euphorbia*	*davidii*	Subils (as E.dentata in Horn et al. 2012)		JN249126	*Chamaesyce*
123	*Euphorbia*	*delicatula*	Boiss.		AF538152	*Chamaesyce*
124	*Euphorbia*	*denisii*	Oudejans		JQ750788	*Chamaesyce*
125	*Euphorbia*	*dentata*	Michx.		JQ750789	*Chamaesyce*
126	*Euphorbia*	*dioscoreoides*		subsp. *attenuata* V. W. Steinm.	JQ750790	*Chamaesyce*
127	*Euphorbia*	*einensis*	G. Will.		JQ750791	*Chamaesyce*
128	*Euphorbia*	*ephedroides*	E. Mey. ex Boiss.		JQ750792	*Chamaesyce*
129	*Euphorbia*	*eriantha*	Benth.		AF538167	*Chamaesyce*
130	*Euphorbia*	*espinosa*	Pax		AF538190	*Chamaesyce*
131	*Euphorbia*	*exilis*	L. C. Leach		JQ750798	*Chamaesyce*
132	*Euphorbia*	*exstipulata*	Engelm.		JQ750793	*Chamaesyce*
133	*Euphorbia*	*florida*	Engelm.		JQ750794	*Chamaesyce*
134	*Euphorbia*	*francoana*	Boiss.		JQ750795	*Chamaesyce*
135	*Euphorbia*	*fulgens*	Karw. ex Klotzsch		AF538154	*Chamaesyce*

Supplementary Table 2 continued. **GenBank accession numbers**. Names and GenBank accession numbers of species sampled.

	Genus	Species	Author	GenBank	Clade
136	*Euphorbia*	*gentilis*	N. E. Br.	JQ750796	*Chamaesyce*
137	*Euphorbia*	*giessii*	L. C. Leach	JQ750797	*Chamaesyce*
138	*Euphorbia*	*glanduligera*	Pax	AF538178	*Chamaesyce*
139	*Euphorbia*	*goetzei*	Pax	AF538185	*Chamaesyce*
140	*Euphorbia*	*goyazensis*	Boiss.	JQ750799	*Chamaesyce*
141	*Euphorbia*	*gradyi*	V. W. Steinm. and Ram.-Roa.	AF538151	*Chamaesyce*
142	*Euphorbia*	*graminea*	Jacq.	KF267329	*Chamaesyce*
143	*Euphorbia*	*guadalajarana*	S. Watson	JQ750802	*Chamaesyce*
144	*Euphorbia*	*gueinzii*	Boiss.	JQ750803	*Chamaesyce*
145	*Euphorbia*	*guerichiana*	Pax	JQ750804	*Chamaesyce*
146	*Euphorbia*	*guiengola*	W. R. Buck and Huft	JQ750805	*Chamaesyce*
147	*Euphorbia*	*gumaroi*	J. Meyran	JQ750806	*Chamaesyce*
148	*Euphorbia*	*gymnoclada*	Boiss.	JQ750807	*Chamaesyce*
149	*Euphorbia*	*herrei*	A. C. White, R. A. Dyer and B. Sloane	JQ750810	*Chamaesyce*
150	*Euphorbia*	*heterophylla*	L.	JQ750811	*Chamaesyce*
151	*Euphorbia*	*hexagona*	Nutt. ex Spreng.	JQ750812	*Chamaesyce*
152	*Euphorbia*	*hindsiana*	Benth.	JQ750813	*Chamaesyce*
153	*Euphorbia*	*hirta*	L.	JQ750815	*Chamaesyce*
154	*Euphorbia*	*hormorrhiza*	Radcl.-Sm.	AF538165	*Chamaesyce*
155	*Euphorbia*	*humayensis*	Brandegee	JQ750816	*Chamaesyce*
156	*Euphorbia*	*humifusa*	Willd.	JQ750817	*Chamaesyce*
157	*Euphorbia*	*hyssopifolia*	L.	JQ750818	*Chamaesyce*
158	*Euphorbia*	*innocua*	L. C. Wheeler	AF538161	*Chamaesyce*
159	*Euphorbia*	*insulana*	Vell.	JQ750819	*Chamaesyce*
160	*Euphorbia*	*ipecacuanhae*	L.	JN249165	*Chamaesyce*
161	*Euphorbia*	*ixtlana*	Huft	JQ750820	*Chamaesyce*

Supplementary Table 2 continued. **GenBank accession numbers**. Names and GenBank accession numbers of species sampled.

	Genus	Species	Author	GenBank	Clade
162	*Euphorbia*	*jaliscensis*	B. L. Rob. and Greenm.	AF538166	*Chamaesyce*
163	*Euphorbia*	*juttae*	Dinter	AF538188	*Chamaesyce*
164	*Euphorbia*	*kabridarensis*	Thulin	JQ750822	*Chamaesyce*
165	*Euphorbia*	*leistneri*	R. H. Archer	JQ750824	*Chamaesyce*
166	*Euphorbia*	*leucocephala*	Lotsy	JN249172	*Chamaesyce*
167	*Euphorbia*	*lycioides*	Boiss.	JQ750826	*Chamaesyce*
168	*Euphorbia*	*macropodoides*	B. L. Rob. and Greenm.	JQ750827	*Chamaesyce*
169	*Euphorbia*	*macropus*	(Klotzsch and Garcke) Boiss.	JQ750828	*Chamaesyce*
170	*Euphorbia*	*maculata*	L.	JQ750829	*Chamaesyce*
171	*Euphorbia*	*marginata*	Pursh	AJ534788	*Chamaesyce*
172	*Euphorbia*	*mexiae*	Standl.	JQ750831	*Chamaesyce*
173	*Euphorbia*	*misella*	S. Watson	AF538160	*Chamaesyce*
174	*Euphorbia*	*misera*	Benth.	JN249181	*Chamaesyce*
175	*Euphorbia*	*mundii*	N. E. Br.	JQ750832	*Chamaesyce*
176	*Euphorbia*	*ocymoidea*	L.	JQ750833	*Chamaesyce*
177	*Euphorbia*	*oerstediana*	(Klotzsch. and Garcke) Boiss.	AF538159	*Chamaesyce*
178	*Euphorbia*	*pediculifera*	Engelm.	JQ750834	*Chamaesyce*
179	*Euphorbia*	*petiolata*	Banks and Sol.	AF538180	*Chamaesyce*
180	*Euphorbia*	*phylloclada*	Boiss.	AF538179	*Chamaesyce*
181	*Euphorbia*	*pinetorum*	(Small) G. L. Webster	JQ750837	*Chamaesyce*
182	*Euphorbia*	*pirottae*	N. Terrac.	AF538186	*Chamaesyce*
183	*Euphorbia*	*plagiantha*	Drake	JQ750838	*Chamaesyce*
184	*Euphorbia*	*planiticola*	D. C. Hassall	JQ750839	*Chamaesyce*
185	*Euphorbia*	*platyclada*	Rauh	AF538187	*Chamaesyce*
186	*Euphorbia*	*polyantha*	Pax	EU022107	*Chamaesyce*
187	*Euphorbia*	*polygonifolia*	L.	JQ750840	*Chamaesyce*

Supplementary Table 2 continued. **GenBank accession numbers**. Names and GenBank accession numbers of species sampled.

	Genus	Species	Author	GenBank	Clade
188	*Euphorbia*	*polyphylla*	Engelm. ex Holz.	JQ750841	*Chamaesyce*
189	*Euphorbia*	*pulcherrima*	Willd. ex Klotzsch	JN249207	*Chamaesyce*
190	*Euphorbia*	*pumicicola*	Huft	AF538164	*Chamaesyce*
191	*Euphorbia*	*quaitensis*	S. Carter	EU022061	*Chamaesyce*
192	*Euphorbia*	*radians*	Benth.	AF538169	*Chamaesyce*
193	*Euphorbia*	*rhombifolia*	Boiss.	JQ750843	*Chamaesyce*
194	*Euphorbia*	*salota*	Leandri	JQ750846	*Chamaesyce*
195	*Euphorbia*	*sarcodes*	Boiss.	JQ750847	*Chamaesyce*
196	*Euphorbia*	*scandens*	Kunth	JQ750848	*Chamaesyce*
197	*Euphorbia*	*scatorhiza*	S. Carter	AF538181	*Chamaesyce*
198	*Euphorbia*	*schlechtendalii*	Boiss.	JQ750849	*Chamaesyce*
199	*Euphorbia*	*sciadophila*	Boiss.	JQ750850	*Chamaesyce*
200	*Euphorbia*	*sessilifolia*	Klotzsch ex Boiss.	JQ750851	*Chamaesyce*
201	*Euphorbia*	*setiloba*	Engelm. ex Torr.	JQ750852	*Chamaesyce*
202	*Euphorbia*	*setosa*	(Boiss.) Mull. Arg.	JQ750853	*Chamaesyce*
203	*Euphorbia*	*sinaloensis*	Brandegee	AF538156	*Chamaesyce*
204	*Euphorbia*	*sonorae*	Rose	JQ750854	*Chamaesyce*
205	*Euphorbia*	*soobyi*	McVaugh	JQ750855	*Chamaesyce*
206	*Euphorbia*	*spartaria*	N. E. Br.	JQ750859	*Chamaesyce*
207	*Euphorbia*	*sphaerorhiza*	Benth.	JQ750860	*Chamaesyce*
208	*Euphorbia*	*spinea*	N. E. Br.	JQ750862	*Chamaesyce*
209	*Euphorbia*	*strigosa*	Hook. and Arn.	AF538163	*Chamaesyce*
210	*Euphorbia*	*subpeltata*	S. Watson	JQ750863	*Chamaesyce*
211	*Euphorbia*	*subreniformis*	S. Watson	JQ750864	*Chamaesyce*
212	*Euphorbia*	*succedanea*	L. C. Wheeler	AF538162	*Chamaesyce*
213	*Euphorbia*	*tannensis*	Spreng.	AF538184	*Chamaesyce*
214	*Euphorbia*	*thymifolia*	L.	JQ750865	*Chamaesyce*
215	*Euphorbia*	*tomentulosa*	S. Watson	JN249236	*Chamaesyce*

Supplementary Table 2 continued. **GenBank accession numbers**. Names and GenBank accession numbers of species sampled.

	Genus	Species	Author	GenBank	Clade
216	*Euphorbia*	*tresmariae*	(Millsp.) Standl.	JQ750866	*Chamaesyce*
217	*Euphorbia*	*xalapensis*	Kunth	JQ750867	*Chamaesyce*
218	*Euphorbia*	*xanti*	Engelm. ex Boiss.	JQ750868	*Chamaesyce*
219	*Euphorbia*	*acanthothamnos*	Heldr. and Sart. ex Boiss.	JQ750756	*Esula*
220	*Euphorbia*	*agraria*	M. Bieb.	KC212433	*Esula*
221	*Euphorbia*	*akenocarpa*	Guss.	JN249098	*Esula*
222	*Euphorbia*	*alaica*	(Porkh.) Porkh.	KC212436	*Esula*
223	*Euphorbia*	*aleppica*	L.	KC212437	*Esula*
224	*Euphorbia*	*amygdaloides*	L.	KC212439	*Esula*
225	*Euphorbia*	*anachoreta*	Svent.	KC212440	*Esula*
226	*Euphorbia*	*angulata*	Jacq.	KC212175	*Esula*
227	*Euphorbia*	*aphylla*	Brouss. ex Willd.	AF538225	*Esula*
228	*Euphorbia*	*arvalis*	subsp. *arvalis* Boiss. and Heldr.	KC212442	*Esula*
229	*Euphorbia*	*aserbajdzhanica*	Bordz.	KC212444	*Esula*
230	*Euphorbia*	*atropurpurea*	Brouss. ex Willd.	KC212445	*Esula*
231	*Euphorbia*	*aucheri*	Boiss.	KC212446	*Esula*
232	*Euphorbia*	*austriaca*	A. Kern.	KC212447	*Esula*
233	*Euphorbia*	*azorica*	Hochst.	KC212448	*Esula*
234	*Euphorbia*	*barrelieri*	Savi	KC212449	*Esula*
235	*Euphorbia*	*berotica*	N. E. Br.	KC212451	*Esula*
236	*Euphorbia*	*biumbellata*	Poir.	KC212452	*Esula*
237	*Euphorbia*	*boetica*	Boiss.	KC212453	*Esula*
238	*Euphorbia*	*borealis*	Baikov	KC212455	*Esula*
239	*Euphorbia*	*bourgeana*	J. Gay ex Boiss.	KC212456	*Esula*
240	*Euphorbia*	*brachycera*	Engelm.	KC212458	*Esula*
241	*Euphorbia*	*briquetii*	Emb. and Maire	KC212459	*Esula*
242	*Euphorbia*	*bungei*	Boiss.	KC212460	*Esula*

Supplementary Table 2 continued. **GenBank accession numbers**. Names and GenBank accession numbers of species sampled.

	Genus	Species	Author		GenBank	Clade
243	*Euphorbia*	*caeladenia*	Boiss.		KC212461	*Esula*
244	*Euphorbia*	*caesia*	Kar. and Kir.		KC212462	*Esula*
245	*Euphorbia*	*calyptrata*	Coss. and Durieu		JN249113	*Esula*
246	*Euphorbia*	*capitulata*	Rchb.		KC212465	*Esula*
247	*Euphorbia*	*carpatica*	Wol.		KC212466	*Esula*
248	*Euphorbia*	*cassia*	Boiss.		KC212467	*Esula*
249	*Euphorbia*	*ceratocarpa*	Ten.		KC212468	*Esula*
250	*Euphorbia*	*characias*		subsp. *characias* L.	KC212469	*Esula*
251	*Euphorbia*	*cheiradenia*	Boiss. and Hohen.		KC212471	*Esula*
252	*Euphorbia*	*commutata*	Engelm. ex A. Gray		KC212472	*Esula*
253	*Euphorbia*	*condylocarpa*	M. Bieb.		KC212473	*Esula*
254	*Euphorbia*	*connata*	Boiss.		KC212475	*Esula*
255	*Euphorbia*	*corallioides*	L.		KC212476	*Esula*
256	*Euphorbia*	*craspedia*	Boiss.		KC212477	*Esula*
257	*Euphorbia*	*cyparissias*	L.		KC212480	*Esula*
258	*Euphorbia*	*cyparissioides*	Pax		KC212481	*Esula*
259	*Euphorbia*	*deltobracteata*	(Porkh.) Porkh.		KC212482	*Esula*
260	*Euphorbia*	*dendroides*	L.		JN249125	*Esula*
261	*Euphorbia*	*densa*	Schrenk		KC212484	*Esula*
262	*Euphorbia*	*densiusculiformis*	(Pazij) Botsch.		KC212487	*Esula*
263	*Euphorbia*	*denticulata*	Lam.		KC212488	*Esula*
264	*Euphorbia*	*depauperata*	Hochst. ex A. Rich.		KC212489	*Esula*
265	*Euphorbia*	*dimorphocaulon*	P. H. Davis		JN249127	*Esula*
266	*Euphorbia*	*discolor*	Ledeb.		KC212493	*Esula*
267	*Euphorbia*	*dracunculoides*		subsp. *inconspicua* (Ball) Maire	KC212494	*Esula*
268	*Euphorbia*	*dulcis*	L.		KC212495	*Esula*
269	*Euphorbia*	*dumalis*	S. Carter		KC212497	*Esula*

Supplementary Table 2 continued. **GenBank accession numbers**. Names and GenBank accession numbers of species sampled.

	Genus	Species	Author	GenBank	Clade
270	*Euphorbia*	*epicyparissias*	(E. Mey. ex Klotzsch and Garke) Boiss.	KC212500	*Esula*
271	*Euphorbia*	*ericoides*	Lam.	JN249134	*Esula*
272	*Euphorbia*	*erubescens*	Boiss.	KC212501	*Esula*
273	*Euphorbia*	*esula*	L.	KC212502	*Esula*
274	*Euphorbia*	*exigua*	L.	KC212506	*Esula*
275	*Euphorbia*	*falcata*	L.	KC212507	*Esula*
276	*Euphorbia*	*fistulosa*	M. S. Khan	KC212509	*Esula*
277	*Euphorbia*	*flavicoma*	DC.	JN249142	*Esula*
278	*Euphorbia*	*fragifera*	Jan	KC212514	*Esula*
279	*Euphorbia*	*franchettii*	B. Fedtsch.	KC212516	*Esula*
280	*Euphorbia*	*furcatifolia*	M. G. Gilbert	KC212517	*Esula*
281	*Euphorbia*	*furcillata*	Kunth	KC212518	*Esula*
282	*Euphorbia*	*gaillardotii*	Boiss. and Blanche	KC212519	*Esula*
283	*Euphorbia*	*genistoides*	P. J. Bergius	KC212520	*Esula*
284	*Euphorbia*	*glabriflora*	Vis.	KC212521	*Esula*
285	*Euphorbia*	*glareosa*	Pall. ex M. Bieb.	KC212522	*Esula*
286	*Euphorbia*	*glauca*	G. Forst.	KC212523	*Esula*
287	*Euphorbia*	*gossypina*	Pax	KC212525	*Esula*
288	*Euphorbia*	*gregersenii*	K. Maly ex Beck	KC212528	*Esula*
289	*Euphorbia*	*grossheimii*	(Porkh.) Porkh.	KC212529	*Esula*
290	*Euphorbia*	*guyoniana*	Boiss. and Reut.	JN249155	*Esula*
291	*Euphorbia*	*gypsicola*	Rech.f. and Aellen	KC212530	*Esula*
292	*Euphorbia*	*haussknechtii*	Boiss.	KC212531	*Esula*
293	*Euphorbia*	*hebecarpa*	Boiss.	KC212532	*Esula*
294	*Euphorbia*	*helioscopia*	L.	KC212533	*Esula*
295	*Euphorbia*	*hercegovina*	Beck	KC212535	*Esula*
296	*Euphorbia*	*herniariifolia*	Willd.	KC212536	*Esula*

Supplementary Table 2 continued. **GenBank accession numbers**. Names and GenBank accession numbers of species sampled.

	Genus	Species	Author	GenBank	Clade
297	*Euphorbia*	*heteradena*	Jaub. and Spach	KC212537	*Esula*
298	*Euphorbia*	*himalayensis*	(Klotzsch) Boiss.	KC212648	*Esula*
299	*Euphorbia*	*hirsuta*	L.	JN249161	*Esula*
300	*Euphorbia*	*hyberna*	L.	KC212538	*Esula*
301	*Euphorbia*	*iberica*	Boiss.	KC212540	*Esula*
302	*Euphorbia*	*illirica*	Lam.	KC212542	*Esula*
303	*Euphorbia*	*inderiensis*	Less. ex Kar. and Kir.	KC212544	*Esula*
304	*Euphorbia*	*isatidifolia*	Lam.	KC212545	*Esula*
305	*Euphorbia*	*isaurica*	M. S. Khan	KC212547	*Esula*
306	*Euphorbia*	*jolkinii*	Boiss.	KC212548	*Esula*
307	*Euphorbia*	*kerneri*	Huter ex A. Kern.	KC212549	*Esula*
308	*Euphorbia*	*kopetdaghi*	(Porkh.) Porkh.	KC212550	*Esula*
309	*Euphorbia*	*lagascae*	Spreng.	KC212551	*Esula*
310	*Euphorbia*	*lamarckii*	Sweet	KC212553	*Esula*
311	*Euphorbia*	*lamprocarpa*	(Porkh.) Porkh.	KC212554	*Esula*
312	*Euphorbia*	*lateriflora*	Schumach.	JN249169	*Esula*
313	*Euphorbia*	*lathyris*	L.	JN249170	*Esula*
314	*Euphorbia*	*lucida*	Waldst. and Kit.	KC212557	*Esula*
315	*Euphorbia*	*lurida*	Engelm.	JN249176	*Esula*
316	*Euphorbia*	*macrocarpa*	Boiss. and Buhse	KC212558	*Esula*
317	*Euphorbia*	*macroceras*	Fisch. and C. A. Mey.	KC212560	*Esula*
318	*Euphorbia*	*macroclada*	Boiss.	KC212562	*Esula*
319	*Euphorbia*	*malleata*	Boiss.	KC212563	*Esula*
320	*Euphorbia*	*matritensis*	Boiss.	KC212564	*Esula*
321	*Euphorbia*	*mauritanica*	L.	JN249179	*Esula*
322	*Euphorbia*	*mazandaranica*	Pahlevani	KC212567	*Esula*
323	*Euphorbia*	*megalatlantica*	Ball	KC212569	*Esula*
324	*Euphorbia*	*mellifera*	Aiton	KC212570	*Esula*

Supplementary Table 2 continued. **GenBank accession numbers**. Names and GenBank accession numbers of species sampled.

	Genus	Species	Author		GenBank	Clade
325	*Euphorbia*	*micractina*	Boiss.		KC212571	*Esula*
326	*Euphorbia*	*microsciadia*	Boiss.		KC212572	*Esula*
327	*Euphorbia*	*minuta*	Loscos and J. Pardo		KC212573	*Esula*
328	*Euphorbia*	*montenegrina*	(Bald.) K. Maly		KC212574	*Esula*
329	*Euphorbia*	*muraltioides*	N. E. Br.		KC212575	*Esula*
330	*Euphorbia*	*myrsinites*	L.		KC212576	*Esula*
331	*Euphorbia*	*natalensis*	Bernh. ex Krauss		KC212577	*Esula*
332	*Euphorbia*	*neilmulleri*	M. C. Johnst.		KC212578	*Esula*
333	*Euphorbia*	*nereidum*	Jahand. and Maire		JN249186	*Esula*
334	*Euphorbia*	*nevadensis*		subsp. *bolosii* Molero and Rovira	KC212580	*Esula*
335	*Euphorbia*	*nicaeensis*	All.		JN249188	*Esula*
336	*Euphorbia*	*niciciana*	Borbas ex Novak		KC212584	*Esula*
337	*Euphorbia*	*nubica*	N. E. Br.		KC212586	*Esula*
338	*Euphorbia*	*oblongata*	Griseb.		KC212587	*Esula*
339	*Euphorbia*	*oblongifolia*	(K. Koch) K. Koch		KC212588	*Esula*
340	*Euphorbia*	*orthoclada*	Baker		JN249191	*Esula*
341	*Euphorbia*	*oxyphylla*	Boiss.		JN249192	*Esula*
342	*Euphorbia*	*palustris*	L.		KC212591	*Esula*
343	*Euphorbia*	*pancicii*	Beck		KC212592	*Esula*
344	*Euphorbia*	*paniculata*		subsp. *paniculata* Desf.	KC212594	*Esula*
345	*Euphorbia*	*paralias*	L.		JN249194	*Esula*
346	*Euphorbia*	*pedroi*	Molero and Rovira		KC212596	*Esula*
347	*Euphorbia*	*peplus*	L.		JN249197	*Esula*
348	*Euphorbia*	*phymatosperma*	Boiss. and Gaill.		KC212599	*Esula*
349	*Euphorbia*	*pinkavana*	M. C. Johnst.		KC212601	*Esula*
350	*Euphorbia*	*pithyusa*	L.		KC212602	*Esula*
351	*Euphorbia*	*platyphyllos*	L.		KC212603	*Esula*

Supplementary Table 2 continued. **GenBank accession numbers**. Names and GenBank accession numbers of species sampled.

	Genus	Species	Author		GenBank	Clade
352	*Euphorbia*	*polycaula*	Boiss. and Hohen.		KC212604	*Esula*
353	*Euphorbia*	*polychroma*	A. Kern.		KC212605	*Esula*
354	*Euphorbia*	*polygalifolia*	Boiss. and Reut.		KC212606	*Esula*
355	*Euphorbia*	*portlandica*	L.		KC212609	*Esula*
356	*Euphorbia*	*pterococca*	Brot.		KC212611	*Esula*
357	*Euphorbia*	*purpurea*	(Raf.) Fernald		KC212613	*Esula*
358	*Euphorbia*	*pyrenaica*	Jord.		KC212614	*Esula*
359	*Euphorbia*	*rapulum*	Kar. and Kir.		KC212616	*Esula*
360	*Euphorbia*	*regis-jubae*	Webb and Berthel.		JN249213	*Esula*
361	*Euphorbia*	*retusa*	Forssk.		JN249213	*Esula*
362	*Euphorbia*	*rhabdotosperma*	Radcl.-Sm.		KC212618	*Esula*
363	*Euphorbia*	*rigida*	M. Bieb.		KC212619	*Esula*
364	*Euphorbia*	*rimarum*	Coss. and Balansa		JN249217	*Esula*
365	*Euphorbia*	*rupestris*	Ledeb.		KC212620	*Esula*
366	*Euphorbia*	*sahendi*	Bornm.		KC212621	*Esula*
367	*Euphorbia*	*salicifolia*	Host		KC212623	*Esula*
368	*Euphorbia*	*schimperi*	C. Presl		KC212624	*Esula*
369	*Euphorbia*	*schimperiana*	Scheele		KC212629	*Esula*
370	*Euphorbia*	*sclerocyathium*	Korovin and Popov		JN249224	*Esula*
371	*Euphorbia*	*sclerophylla*	Boiss.		KC212630	*Esula*
372	*Euphorbia*	*segetalis*		var. *pinea* (L.) Lange	KC212600	*Esula*
373	*Euphorbia*	*seguieriana*	Neck.		KC212631	*Esula*
374	*Euphorbia*	*semivillosa*	(Porkh.) Krylov		KC212632	*Esula*
375	*Euphorbia*	*serrata*	L.		KC212633	*Esula*
376	*Euphorbia*	*sojakii*	(Chrtek and Krisa) Dubovik		KC212634	*Esula*
377	*Euphorbia*	*spathulata*	Lam.		JN249229	*Esula*
378	*Euphorbia*	*spinidens*	Bornm. ex Porkh.		KC212638	*Esula*

Supplementary Table 2 continued. **GenBank accession numbers**. Names and GenBank accession numbers of species sampled.

	Genus	Species	Author	GenBank	Clade
379	*Euphorbia*	*spinosa*	L.	KC212641	*Esula*
380	*Euphorbia*	*squamigera*	Loisel.	KC212642	*Esula*
381	*Euphorbia*	*stepposa*	Zoz	KC212643	*Esula*
382	*Euphorbia*	*stolonifera*	Marloth ex A. C. White	KC212644	*Esula*
383	*Euphorbia*	*stracheyi*	Boiss.	KC212645	*Esula*
384	*Euphorbia*	*stricta*	L.	KC212649	*Esula*
385	*Euphorbia*	*stygiana*	H. C. Watson	KC212651	*Esula*
386	*Euphorbia*	*sulcata*	Lens ex Loisel.	KC212652	*Esula*
387	*Euphorbia*	*sultan-hassei*	A. Strid and al.	KC212653	*Esula*
388	*Euphorbia*	*szovitsii*	Fisch and C. A. Mey.	KC212655	*Esula*
389	*Euphorbia*	*talastavica*	(Porkh.) Porkh.	KC212657	*Esula*
390	*Euphorbia*	*teheranica*	Boiss.	KC212658	*Esula*
391	*Euphorbia*	*terracina*	L.	KC212660	*Esula*
392	*Euphorbia*	*texana*	Boiss.	KC212661	*Esula*
393	*Euphorbia*	*transtagana*	Boiss.	KC212662	*Esula*
394	*Euphorbia*	*triflora*	Schott, Nyman and Kotschy	KC212663	*Esula*
395	*Euphorbia*	*tshuiensis*	(Porkh.) Serg. ex Krylov	KC212664	*Esula*
396	*Euphorbia*	*tuckeyana*	Steud. ex Webb	KC212667	*Esula*
397	*Euphorbia*	*turczaninowii*	Kar. and Kir.	KC212668	*Esula*
398	*Euphorbia*	*uliginosa*	Welw. ex Boiss.	KC212669	*Esula*
399	*Euphorbia*	*usambarica*	Pax	KC212670	*Esula*
400	*Euphorbia*	*valerianifolia*	Lam.	KC212671	*Esula*
401	*Euphorbia*	*valliniana*	Belli	KC212672	*Esula*
402	*Euphorbia*	*variabilis*	Ces.	KC212673	*Esula*
403	*Euphorbia*	*velenovskyi*	Bornm.	KC212674	*Esula*
404	*Euphorbia*	*veneris*	M. S. Khan	KC212675	*Esula*
405	*Euphorbia*	*verrucosa*	L.	KC212676	*Esula*
406	*Euphorbia*	*virgata*	Waldst. and Kit.	KC212678	*Esula*

Supplementary Table 2 continued. **GenBank accession numbers**. Names and GenBank accession numbers of species sampled.

	Genus	Species	Author	GenBank	Clade
407	*Euphorbia*	*walachii*	Hook.f.	KC212683	*Esula*
408	*Euphorbia*	*x popovii*	Rotschild	KC212608	*Esula*
409	*Euphorbia*	*abdelkuri*	Balf.f.	KC019836	*Euphorbia*
410	*Euphorbia*	*abyssinica*	J. F. Gmel.	KC019793	*Euphorbia*
411	*Euphorbia*	*aff. retrospina*		KC019757	*Euphorbia*
412	*Euphorbia*	*alfredii*	Rauh	KC019827	*Euphorbia*
413	*Euphorbia*	*alluaudii*	Drake	KC019746	*Euphorbia*
414	*Euphorbia*	*ammak*	Schweinf.	KC019820	*Euphorbia*
415	*Euphorbia*	*antiquorum*	L.	KC019777	*Euphorbia*
416	*Euphorbia*	*arbuscula*	Balf. f.	JN249105	*Euphorbia*
417	*Euphorbia*	*avasmontana*	Dinter	KC019783	*Euphorbia*
418	*Euphorbia*	*beharensis*	Leandri	KC019832	*Euphorbia*
419	*Euphorbia*	*bisellenbeckii*	Bruyns	KC019810	*Euphorbia*
420	*Euphorbia*	*boophthona*	C. A. Gardner	KC019775	*Euphorbia*
421	*Euphorbia*	*bougheyi*	L. C. Leach	KC019814	*Euphorbia*
422	*Euphorbia*	*bracteata*	Jacq.	KC019845	*Euphorbia*
423	*Euphorbia*	*breviarticulata*	Pax	JQ750771	*Euphorbia*
424	*Euphorbia*	*bussei*	Pax	KC019796	*Euphorbia*
425	*Euphorbia*	*caducifolia*	Haines	KC019809	*Euphorbia*
426	*Euphorbia*	*caerulscens*	Haw.	KC019782	*Euphorbia*
427	*Euphorbia*	*calyculata*	Kunth	AF538221	*Euphorbia*
428	*Euphorbia*	*capsaintemariensis*	(Rauh) Cremers	KC019760	*Euphorbia*
429	*Euphorbia*	*cestrifolia*	Kunth	AF538213	*Euphorbia*
430	*Euphorbia*	*clivicola*	R. A. Dyer	KC019855	*Euphorbia*
431	*Euphorbia*	*comosa*	Vell.	JN249118	*Euphorbia*
432	*Euphorbia*	*confinalis*	R. A. Dyer	KC019849	*Euphorbia*
433	*Euphorbia*	*cooperi*	N. E. Br.	KC019858	*Euphorbia*
434	*Euphorbia*	*croizatii*	Leandri	KC019761	*Euphorbia*

Supplementary Table 2 continued. **GenBank accession numbers**. Names and GenBank accession numbers of species sampled.

	Genus	Species	Author	GenBank	Clade
435	*Euphorbia*	*cubensis*	Boiss.	EF653259	*Euphorbia*
436	*Euphorbia*	*curvirama*	R. A. Dyer	KC019791	*Euphorbia*
437	*Euphorbia*	*cylindrifolia*	Marn.-Lap. and Rauh	KC019834	*Euphorbia*
438	*Euphorbia*	*damarana*	L. C. Leach	KC019866	*Euphorbia*
439	*Euphorbia*	*decaryi*	Guillaumin	KC019831	*Euphorbia*
440	*Euphorbia*	*decidua*	P. R. O. Bally and L. C. Leach	KC019864	*Euphorbia*
441	*Euphorbia*	*desmondii*	Keay and Milne-Redh.	KC019871	*Euphorbia*
442	*Euphorbia*	*didiereoides*	Denis ex Leandri	KC019877	*Euphorbia*
443	*Euphorbia*	*drupifera*	Thonn.	JN249131	*Euphorbia*
444	*Euphorbia*	*elegantissima*	P. R. O. Bally and S. Carter	KC019771	*Euphorbia*
445	*Euphorbia*	*elodes*	Boiss.	KC019779	*Euphorbia*
446	*Euphorbia*	*enormis*	N. E. Br.	KC019816	*Euphorbia*
447	*Euphorbia*	*evansii*	Pax	KC019788	*Euphorbia*
448	*Euphorbia*	*famatamboay*	F. Friedmann and Cremers	KC019763	*Euphorbia*
449	*Euphorbia*	*fanshawei*	L. C. Leach	KC019865	*Euphorbia*
450	*Euphorbia*	*fiherenensis*	Poiss.	KC019749	*Euphorbia*
451	*Euphorbia*	*fractiflexa*	S. Carter and J. R. I. Wood	KC019870	*Euphorbia*
452	*Euphorbia*	*francoisii*	Leandri	KC019841	*Euphorbia*
453	*Euphorbia*	*fruticosa*	Forssk.	KC019812	*Euphorbia*
454	*Euphorbia*	*germainii*	Phil.	AF538205	*Euphorbia*
455	*Euphorbia*	*grandicornis*	Goebel ex N. E. Br.	KC019813	*Euphorbia*
456	*Euphorbia*	*grandidens*	Haw.	KC019785	*Euphorbia*
457	*Euphorbia*	*griseola*	Pax	KC019819	*Euphorbia*
458	*Euphorbia*	*groenewaldii*	R. A. Dyer	KC019854	*Euphorbia*
459	*Euphorbia*	*guentheri*	(Pax) Bruyns	KC019803	*Euphorbia*
460	*Euphorbia*	*gymnonota*	Urb.	JQ750808	*Euphorbia*
461	*Euphorbia*	*haeleeleana*	Herbst	KC019876	*Euphorbia*

Supplementary Table 2 continued. **GenBank accession numbers**. Names and GenBank accession numbers of species sampled.

	Genus	Species	Author	GenBank	Clade
462	*Euphorbia*	*hedyotoides*	N. E. Br.	JN249157	*Euphorbia*
463	*Euphorbia*	*helenae*	Urb.	EF653261	*Euphorbia*
464	*Euphorbia*	*heterochroma*	Pax	KC019797	*Euphorbia*
465	*Euphorbia*	*heterodoxa*	Mull. Arg.	KC019859	*Euphorbia*
466	*Euphorbia*	*heteropoda*	Pax	KC019807	*Euphorbia*
467	*Euphorbia*	*heterospina*	S. Carter	KC019847	*Euphorbia*
468	*Euphorbia*	*hinkleyorum*	I. M. Johnst.	JQ750814	*Euphorbia*
469	*Euphorbia*	*hoffmanniana*	(Klotzsch and Garcke) Boiss.	AF538211	*Euphorbia*
470	*Euphorbia*	*horombensis*	Ursch and Leandri	KC019768	*Euphorbia*
471	*Euphorbia*	*iharanae*	Rauh	KC019840	*Euphorbia*
472	*Euphorbia*	*ingens*	E. Mey. ex Boiss.	KC019857	*Euphorbia*
473	*Euphorbia*	*intisy*	Drake	KC019748	*Euphorbia*
474	*Euphorbia*	*invenusta*	(N. E. Br.) Bruyns	KC019805	*Euphorbia*
475	*Euphorbia*	*kamponii*	Rauh and Petignat	KC019755	*Euphorbia*
476	*Euphorbia*	*keithii*	R. A. Dyer	KC019786	*Euphorbia*
477	*Euphorbia*	*lacei*	Craib	KC019776	*Euphorbia*
478	*Euphorbia*	*lactea*	Haw.	KC019821	*Euphorbia*
479	*Euphorbia*	*lactiflua*	Phil.	AF538219	*Euphorbia*
480	*Euphorbia*	*lagunillarum*	Croizat	KC019874	*Euphorbia*
481	*Euphorbia*	*ledienii*	A. Berger	KC019852	*Euphorbia*
482	*Euphorbia*	*lenewtonii*	S. Carter	KC019842	*Euphorbia*
483	*Euphorbia*	*limpopoana*	L. C. Leach ex S. Carter	KC019851	*Euphorbia*
484	*Euphorbia*	*lividiflora*	L. C. Leach	KC019869	*Euphorbia*
485	*Euphorbia*	*lomelii*	V. W. Steinm.	JN249174	*Euphorbia*
486	*Euphorbia*	*longispina*	Chiov.	KC019862	*Euphorbia*
487	*Euphorbia*	*lophogona*	Lam.	KC019762	*Euphorbia*
488	*Euphorbia*	*micracantha*	Boiss.	KC019817	*Euphorbia*

Supplementary Table 2 continued. **GenBank accession numbers**. Names and GenBank accession numbers of species sampled.

	Genus	Species	Author		GenBank	Clade
489	*Euphorbia*	*milii*	Des Moul.		JN249180	*Euphorbia*
490	*Euphorbia*	*munizii*	Borhidi		EF653262	*Euphorbia*
491	*Euphorbia*	*neoarborescens*	Bruyns		KC019839	*Euphorbia*
492	*Euphorbia*	*neococcinea*	Bruyns		KC019806	*Euphorbia*
493	*Euphorbia*	*neohumbertii*	Boiteau		KC019833	*Euphorbia*
494	*Euphorbia*	*neoreflexa*	Bruyns		KC019843	*Euphorbia*
495	*Euphorbia*	*neorubella*	Bruyns		KC019804	*Euphorbia*
496	*Euphorbia*	*neospinescens*	Bruyns		KC019811	*Euphorbia*
497	*Euphorbia*	*neriifolia*	L.		KC019772	*Euphorbia*
498	*Euphorbia*	*pachysantha*	Baill.		JN249193	*Euphorbia*
499	*Euphorbia*	*pedilanthoides*	Denis		KC019808	*Euphorbia*
500	*Euphorbia*	*peperomioides*	Boiss.		KC019780	*Euphorbia*
501	*Euphorbia*	*persistentifolia*	L. C. Leach		KC019868	*Euphorbia*
502	*Euphorbia*	*pervilleana*	Baill.		JQ750835	*Euphorbia*
503	*Euphorbia*	*phosphorea*	Mart.		JQ750836	*Euphorbia*
504	*Euphorbia*	*plumerioides*	Teijsm. ex Hassk.		KC019848	*Euphorbia*
505	*Euphorbia*	*podocarpifolia*	Urb.		EF653263	*Euphorbia*
506	*Euphorbia*	*portulacoides*	L.	subsp. *collina* (Phil. Croizat)	JN249204	*Euphorbia*
507	*Euphorbia*	*pseudoburuana*	P. R. O. Bally and S. Carter		KC019867	*Euphorbia*
508	*Euphorbia*	*pseudocactus*	A. Berger		KC019787	*Euphorbia*
509	*Euphorbia*	*pseudomollis*	Bruyns		KC019799	*Euphorbia*
510	*Euphorbia*	*pseudotrinervis*	Bruyns		KC019844	*Euphorbia*
511	*Euphorbia*	*pteroneura*	A. Berger		KC019815	*Euphorbia*
512	*Euphorbia*	*punicea*	Sw.		JN249208	*Euphorbia*
513	*Euphorbia*	*ramipressa*	Croizat		KC019824	*Euphorbia*
514	*Euphorbia*	*randrianjohanyi*	Haevermans and Labat		KC019751	*Euphorbia*
515	*Euphorbia*	*rauhii*	Haevermans and Labat		KC019765	*Euphorbia*

Supplementary Table 2 continued. **GenBank accession numbers**. Names and GenBank accession numbers of species sampled.

	Genus	Species	Author	GenBank	Clade
516	*Euphorbia*	*resinifera*	O. Berg	JN249214	*Euphorbia*
517	*Euphorbia*	*rhizophora*	(P. R. O. Bally) Bruyns	KC019802	*Euphorbia*
518	*Euphorbia*	*robecchii*	Pax	KC019825	*Euphorbia*
519	*Euphorbia*	*rosescens*	E. L. Bridges and Orzell	KC019774	*Euphorbia*
520	*Euphorbia*	*rossii*	Rauh and Buchloh	KC019830	*Euphorbia*
521	*Euphorbia*	*royleana*	Boiss.	JQ750845	*Euphorbia*
522	*Euphorbia*	*rubella*	Pax	AF538204	*Euphorbia*
523	*Euphorbia*	*sapinii*	De Wild.	KC019872	*Euphorbia*
524	*Euphorbia*	*seibanica*	Lavranos and Gifri	KC019863	*Euphorbia*
525	*Euphorbia*	*sekukuniensis*	R. A. Dyer	KC019784	*Euphorbia*
526	*Euphorbia*	*sinclairiana*	Benth.	AF538217	*Euphorbia*
527	*Euphorbia*	*sipolisii*	N. E. Br.	KC019778	*Euphorbia*
528	*Euphorbia*	*stellata*	Willd.	KC019789	*Euphorbia*
529	*Euphorbia*	*stenoclada*	Baill.	KC019750	*Euphorbia*
530	*Euphorbia*	*sudanica*	A. Chev.	KC019818	*Euphorbia*
531	*Euphorbia*	*tanaensis*	P. R. O. Bally and S. Carter	KC019798	*Euphorbia*
532	*Euphorbia*	*tanquahuete*	Sesse and Moc.	AF538224	*Euphorbia*
533	*Euphorbia*	*teke*	Schweinf. ex Pax	KC019835	*Euphorbia*
534	*Euphorbia*	*tetragona*	Haw.	KC019781	*Euphorbia*
535	*Euphorbia*	*thinophila*	Phil.	AF538218	*Euphorbia*
536	*Euphorbia*	*tirucalli*	L.	KC019850	*Euphorbia*
537	*Euphorbia*	*tithymaloides*	L.	KC019745	*Euphorbia*
538	*Euphorbia*	*umbellata*	(Pax) Bruyns	JN249237	*Euphorbia*
539	*Euphorbia*	*umbelliformis*	(Urb. and Ekman) V. W. Steinm. and P. E. Berry	EF653258	*Euphorbia*
540	*Euphorbia*	*unispina*	N. E. Br.	JN249239	*Euphorbia*
541	*Euphorbia*	*vajravelui*	Binojk. and N. P. Balakr.	KC019770	*Euphorbia*
542	*Euphorbia*	*venenifica*	Tremaux ex Kotschy	JN249240	*Euphorbia*

Supplementary Table 2 continued. **GenBank accession numbers**. Names and GenBank accession numbers of species sampled.

	Genus	Species	Author		GenBank	Clade
543	*Euphorbia*	*viguieri*	Denis		KC019828	*Euphorbia*
544	*Euphorbia*	*weberbaueri*	Mansf.		AF538212	*Euphorbia*
545	*Euphorbia*	*xylophylloides*	Brongn. ex Lem.		KC019826	*Euphorbia*
546	*Calycopeplus*	*casurinoides*	L. S. Sm.		JN249080	*Outgroup*
547	*Gymnanthes*	*cf. albicans*	(Griseb.) Urb.		JN249083	*Outgroup*
548	*Microstachys*	*chamaelea*	(L.) Mull. Arg.		JN249088	*Outgroup*
549	*Neoguillauminia*	*cleopatra*	(Baill.) Croizat		JN249090	*Outgroup*
550	*Hura*	*crepitans*	L.		JN249085	*Outgroup*
551	*Senefelderopsis*	*croizatii*	Steyerm.		JN249091	*Outgroup*
552	*Dichostemma*	*glaucescens*	Pierre		JN249082	*Outgroup*
553	*Maprounea*	*guianensis*	Aubl.		JN249087	*Outgroup*
554	*Colliguaja*	*integerrima*	Gillies and Hook.		JN249081	*Outgroup*
555	*Bonania*	*microphylla*	Urb.		JN249079	*Outgroup*
556	*Homalanthus*	*nutans*	(G. Forst.) Guill.		JN249084	*Outgroup*
557	*Anthostema*	*senegalense*	A. Juss.		JN249078	*Outgroup*
558	*Stillingia*	*sylvatica*	L.	subsp. *tenuis* (Small) D. J. Rogers	JN249092	*Outgroup*
559	*Mabea*	*taquari*	Aubl.		JN249086	*Outgroup*
560	*Nealchornea*	*yapurensis*	Huber		JN249089	*Outgroup*

Supplementary Table 3. Re-classification of EBDCS *vertebrate poisons*. Re-classification of Economic Botany Data Collection Standard (EBDCS) category *vertebrate poisons* into categories of *inflammatory response*.

EBDCS subcategories				*inflammatory response*
Mammals	humans (126)	Abnormalities (1)	oedemas (1)	possible
Mammals	humans (126)	Digestive System Disorders (12)	purgative (4)	no
Mammals	humans (126)	Digestive System Disorders (12)	emetic (2)	no
Mammals	humans (126)	Digestive System Disorders (12)	vomiting (1)	no
Mammals	humans (126)	Digestive System Disorders (12)	diarrhoea (1)	no
Mammals	humans (126)	Digestive System Disorders (12)	death (2)	no
Mammals	humans (126)	Digestive System Disorders (12)	weight loss (1)	no
Mammals	humans (126)	Digestive System Disorders (12)	milk sickness (1)	no
Mammals	humans (126)	Genitourinary System Disorders (1)	renal damage (1)	no
Mammals	humans (126)	Inflammation (7)		possible
Mammals	humans (126)	Injuries (10)	haemorrhages (2)	no
Mammals	humans (126)	Injuries (10)	blisters (8)	possible
Mammals	humans (126)	Mental Disorders (1)	delirium (1)	no
Mammals	humans (126)	Pain (1)		possible
Mammals	humans (126)	Pregnancy/Birth/Puerperium Disorders (2)	abortifacient (2)	no
Mammals	humans (126)	Sensory System Disorders (11)	blindness (9)	no
Mammals	humans (126)	Sensory System Disorders (11)	unspecified (2)	no
Mammals	humans (126)	Skin/Subcutaneous Cellular Tissue Disorders (20)	rashes (1)	possible
Mammals	humans (126)	Skin/Subcutaneous Cellular Tissue Disorders (20)	irritation (11)	possible
Mammals	humans (126)	Skin/Subcutaneous Cellular Tissue Disorders (20)	hair loss (1)	possible
Mammals	humans (126)	Skin/Subcutaneous Cellular Tissue Disorders (20)	dermatitis (7)	possible
Mammals	humans (126)	Unspecified (60)	unspecified or death	unknown

Supplementary Table 4. Re-classification of EBDCS *medicines*. Re-classification of Economic Botany Data Collection Standard (EBDCS) category *medicines* into categories of *inflammatory response*.

EBDCS subcategories		*inflammatory response*
Abnormalities (37)	oedemas (35)	possible
Abnormalities (37)	lesions (1)	no
Abnormalities (37)	fistula (1)	no
Blood System Disorders (4)	purify blood (2) + unspecified (2)	no + possible (spleen enlargement), no
Circulatory System Disorders (8)	heart disease (5)	no
Circulatory System Disorders (8)	hypertension (3)	no
Digestive System Disorders (143)	purgative (53)	no
Digestive System Disorders (143)	emetic (19)	no
Digestive System Disorders (143)	laxative (8)	no
Digestive System Disorders (143)	diarrhoea (16)	no
Digestive System Disorders (143)	indigestion (5)	no
Digestive System Disorders (143)	constipation (7)	no
Digestive System Disorders (143)	colic (6)	no
Digestive System Disorders (143)	caries (7)	no
Digestive System Disorders (143)	biliousness (1)	no
Digestive System Disorders (143)	bloat (1)	no
Digestive System Disorders (143)	choleretic (1)	no
Digestive System Disorders (143)	carminative (1)	no
Digestive System Disorders (143)	stercorrhagia (1)	no
Digestive System Disorders (143)	jaundice (7)	no
Digestive System Disorders (143)	unspecified (10)	unknown, no (liver disorder, tooth loosening, gargle), possible (dental use to treat affected gums)
Endocrine System Disorders (3)	diabetes mellitus	no
Genitourinary System Disorders (36)	bladder stones (1)	no
Genitourinary System Disorders (36)	chyluria (1)	no
Genitourinary System Disorders (36)	small breasts (1)	no

Supplementary Table 4 continued. **Re-classification of EBDCS *medicines***. Re-classification of Economic Botany Data Collection Standard (EBDCS) category *medicines* into categories of *inflammatory response*.

EBDCS subcategories		*inflammatory response*
Genitourinary System Disorders (36)	aphrodisiac (1)	no
Genitourinary System Disorders (36)	diuretic (4)	no
Genitourinary System Disorders (36)	emmenagogue (1)	no
Genitourinary System Disorders (36)	blennorhagia (2)	possible
Genitourinary System Disorders (36)	dysuria (3)	possible
Genitourinary System Disorders (36)	female infertility (2)	no
Genitourinary System Disorders (36)	male infertility (2)	no
Genitourinary System Disorders (36)	haematuria (6)	possible
Genitourinary System Disorders (36)	impotence (1)	no
Genitourinary System Disorders (36)	leukorrhoea (2)	no
Genitourinary System Disorders (36)	oliguria (1)	no
Genitourinary System Disorders (36)	unspecified (8)	unknown, no (ailments of the urinary tract)
Infections/Infestations (75)	infestations, arthropod infestations (5)	possible
Infections/Infestations (75)	infections, fever (7)	possible
Infections/Infestations (75)	infections, microbial infections, bacterial infections (27)	possible
Infections/Infestations (75)	infections, microbial infections, fungal infections (4)	possible
Infections/Infestations (75)	infections, microbial infections, viral infections (8)	possible
Infections/Infestations (75)	infections, parasitic infections (19)	possible
Infections/Infestations (75)	infections, unspecified (5)	possible
Inflammation (47)	digestive system, intestine (22), gums (1), small intestine (1), unspecified (1)	possible
Inflammation (47)	genitourinary system, breasts (2), kidneys (1)	possible

Supplementary Table 4 continued. **Re-classification of EBDCS *medicines***. Re-classification of Economic Botany Data Collection Standard (EBDCS) category *medicines* into categories of *inflammatory response*.

EBDCS subcategories		*inflammatory response*
Inflammation (47)	muscular-skeletal system, limbs (1), bones (1), soft tissues (2)	possible
Inflammation (47)	respiratory system, bronchi (9), respiratory mucosae (1), unspecified (1)	possible
Inflammation (47)	sensory system, conjuctivae, (4)	possible
Injuries (60)	abscesses (3)	possible
Injuries (60)	blisters (2)	possible
Injuries (60)	burns (1)	possible
Injuries (60)	haemorrhages (26)	no
Injuries (60)	injuries (1)	no
Injuries (60)	wounds (26)	possible
Injuries (60)	unspecified (1)	possible
Mental Disorders (7)	hallucinogenic (1)	no
Mental Disorders (7)	narcotic (1)	no
Mental Disorders (7)	alcohol dependence (3)	no
Mental Disorders (7)	sleep disorders, hypnotic (1)	no
Mental Disorders (7)	unspecified (1)	no
Metabolic System Disorders (10)	gout (1)	possible
Metabolic System Disorders (10)	diaphoretic (9)	no
Muscular-Skeletal System Disorders (12)	rheumatism (7)	possible
Muscular-Skeletal System Disorders (12)	spasms (1)	no
Muscular-Skeletal System Disorders (12)	sprains (2)	possible
Muscular-Skeletal System Disorders (12)	asthenia (1)	possible
Muscular-Skeletal System Disorders (12)	lumbago (1)	possible
Neoplasms (15)	unspecified neoplasms (14)	possible
Neoplasms (15)	malignant neoplasms (1)	possible
Nervous System Disorders (7)	stimulant (4)	no

Supplementary Table 4 continued. **Re-classification of EBDCS *medicines***. Re-classification of Economic Botany Data Collection Standard (EBDCS) category *medicines* into categories of *inflammatory response*.

EBDCS subcategories		*inflammatory response*
Nervous System Disorders (7)	paralysis (2)	no
Nervous System Disorders (7)	unspecified (1)	unknown
Nutritional Disorders (10)	malnutrition (1)	no
Nutritional Disorders (10)	tonic (8)	no
Nutritional Disorders (10)	vitamin D deficiency (1)	no
Pain (31)	muscular-skeletal system, chest (3), head (3), body (1)	possible
Pain (31)	digestive system, teeth (10), stomach (5)	possible
Pain (31)	nervous system, nerves (1)	no
Pain (31)	respiratory system, pharynx (1)	possible
Pain (31)	sensory system, ears (1)	possible
Pain (31)	skin/subcutaenous cellular tissue disorders, skin (1)	possible
Pain (31)	unspecified (5)	unknown
Poisonings (16)	bites and stings (12)	no
Poisonings (16)	antihistaminic (1)	possible
Poisonings (16)	medicine poisoning (1)	unknown
Poisonings (16)	unspecified (2)	no
Pregnancy/Birth/Puerperium Disorders (23)	lactation stimulant (15)	no
Pregnancy/Birth/Puerperium Disorders (23)	labour induction (1)	no
Pregnancy/Birth/Puerperium Disorders (23)	postpartum depurant (1)	no
Pregnancy/Birth/Puerperium Disorders (23)	unspecified (6)	unknown
Respiratory System Disorders (40)	bronchial affections (3)	possible
Respiratory System Disorders (40)	asthma (22)	possible
Respiratory System Disorders (40)	expectorant (1)	no
Respiratory System Disorders (40)	breathlessness (1)	possible

Supplementary Table 4 continued. **Re-classification of EBDCS *medicines***. Re-classification of Economic Botany Data Collection Standard (EBDCS) category *medicines* into categories of *inflammatory response*.

EBDCS subcategories		*inflammatory response*
Respiratory System Disorders (40)	pneumonia (1)	possible
Respiratory System Disorders (40)	coughs (10)	possible
Respiratory System Disorders (40)	unspecified (2)	no (errhine), unknown
Sensory System Disorders (21)	ear troubles (1)	no
Sensory System Disorders (21)	remove pus inside ear (1)	no
Sensory System Disorders (21)	deafness (1)	no
Sensory System Disorders (21)	eye disorders (14)	no
Sensory System Disorders (21)	remove corneal opacities (1)	possible
Sensory System Disorders (21)	blindness (1)	no
Sensory System Disorders (21)	cataracts (2)	possible
Skin/Subcutaneous Cellular Tissue Disorders (99)	emollient (1)	no
Skin/Subcutaneous Cellular Tissue Disorders (99)	disinfectant (1)	no
Skin/Subcutaneous Cellular Tissue Disorders (99)	astringent (6)	no
Skin/Subcutaneous Cellular Tissue Disorders (99)	antiseptic (1)	no
Skin/Subcutaneous Cellular Tissue Disorders (99)	whitlows (1)	possible
Skin/Subcutaneous Cellular Tissue Disorders (99)	warts (18)	possible
Skin/Subcutaneous Cellular Tissue Disorders (99)	ulcers (3)	possible
Skin/Subcutaneous Cellular Tissue Disorders (99)	sunburn (1)	possible
Skin/Subcutaneous Cellular Tissue Disorders (99)	sores (13)	possible
Skin/Subcutaneous Cellular Tissue Disorders (99)	rashes (4)	possible
Skin/Subcutaneous Cellular Tissue Disorders (99)	pustules (1)	possible
Skin/Subcutaneous Cellular Tissue Disorders (99)	psoriasis (1)	possible
Skin/Subcutaneous Cellular Tissue Disorders (99)	itching (5)	possible
Skin/Subcutaneous Cellular Tissue Disorders (99)	irritation (1)	possible
Skin/Subcutaneous Cellular Tissue Disorders (99)	hair loss (1)	possible
Skin/Subcutaneous Cellular Tissue Disorders (99)	eczema (6)	possible
Skin/Subcutaneous Cellular Tissue Disorders (99)	dermatitis (2)	possible

Supplementary Table 4 continued. **Re-classification of EBDCS *medicines***. Re-classification of Economic Botany Data Collection Standard (EBDCS) category *medicines* into categories of *inflammatory response*.

EBDCS subcategories		*inflammatory response*
Skin/Subcutaneous Cellular Tissue Disorders (99)	carbuncles (6)	no
Skin/Subcutaneous Cellular Tissue Disorders (99)	calluses (1)	no
Skin/Subcutaneous Cellular Tissue Disorders (99)	boils (2)	no
Skin/Subcutaneous Cellular Tissue Disorders (99)	acne (2)	no
Skin/Subcutaneous Cellular Tissue Disorders (99)	rubefacient (1)	possible
Skin/Subcutaneous Cellular Tissue Disorders (99)	remove thorns (1)	no
Skin/Subcutaneous Cellular Tissue Disorders (99)	moles (1)	possible
Skin/Subcutaneous Cellular Tissue Disorders (99)	freckles (1)	possible
Skin/Subcutaneous Cellular Tissue Disorders (99)	unspecified (18)	unknown
Unspecified Medicinal Disorders (78)		unknown

References

1. Katoh, K. & Standley, D. M. MAFFT multiple sequence alignment software version 7: improvements in performance and usability. Mol. Biol. Evol. **30,** 772-780 (2013).

2. Maddison, W. P. & Maddison, D. R. Mesquite: a modular system for evolutionary analaysis. Version 3.01. http://mesquiteproject.org (2014).

3. Guindon, S. & Gascuel, O. A simple, fast and accurate method to estimate large phylogenies by maximum-likelihood. Systematic Biology **52,** 696-704 (2003).

4. Darriba, D., Taboada, G. L., Doallo, R. & Posada, D. jModelTest 2: more models, new heuristics and parallel computing. Nat. Meth. **9,** 772 (2012).

5. Akaike, H. 1974. A new look at the statistical model identification. IEEE T. Automat. Contr. **19,** 716-723 (1974).

6. Yang, Z. & Rannala, B. Bayesian phylogenetic inference using DNA sequences a Markov Chain Monte Carlo method. Mol. Biol. Evol. **14,** 717-724 (1997).

7. Ronquist, F. et al. MrBayes 3.2: Efficient Bayesian Phylogenetic Inference and Model Choice Across a Large Model Space. Syst. Biol. **61,** 539-542 (2012).

8. Rambaut, A., Suchard, M. A., Xie, D. & Drummond, A. J. Tracer v1.6. Available from http://beast.bio.ed.ac.uk/Tracer (2014).

9. Ernst, M. et al. Global medicinal uses of *Euphorbia* L. (Euphorbiaceae). J. Ethnopharmacol. **176,** 90-101 (2015).

10. Fritz, S. A. & Purvis, A. Selectivity in mammalian extinction risk and threat types: A new measure of phylogenetic signal strength in binary traits. Conserv. Biol. **24,** 1042-1051 (2010).

11. Paradis, E., Claude, J. & Strimmer, K. APE: analyses of phylogenetics and evolution in R language. Bioinformatics **20,** 289-290 (2004).

12. Luke, H. J., Weir, J. T., Brock, C. D., Glor, R. E., Challenger, W. GEIGER: investigating evolutionary radiations. Bioinformatics **24,** 129-131 (2008).

13. Wickham, H. The Split-Apply-Combine strategy for data analysis. J. Stat. Softw. **1,** 1-29 (2011).

14. Yang, Y. et al. Molecular phylogenetics and classification of *Euphorbia* subgenus *Chamaesyce* (Euphorbiaceae). TAXON **61,** 764-789 (2012).

15. Dorsey, B. L. et al. Phylogenetics, morphological evolution, and classification of *Euphorbia* subgenus *Euphorbia* (Euphorbiaceae). TAXON **62,** 291-315 (2013).

16. Peirson, J. A., Bruyns, P. V., Riina, R., Morawetz, J. J. & Berry, P. E. A molecular phylogeny and classification of the largely succulent and mainly African *Euphorbia* subg. *Athymalus* (Euphorbiaceae). TAXON **62,** 1178-1199 (2013).

17. Riina, R. et al. A worldwide molecular phylogeny and classification of the leafy spurges, *Euphorbia* subgenus *Esula* (Euphorbiaceae). TAXON **62,** 316-342 (2013).
